# Synthesis of Novel Gefitinib-Conjugated 1,2,3-Triazole Derivatives and Their Effect of Inducing DNA Damage and Apoptosis in Tumor Cells

**DOI:** 10.3390/molecules29225438

**Published:** 2024-11-18

**Authors:** Junfei Wu, Xu Huang, Shan Lu, Ziyi Wang, Longfei Mao, Sanqiang Li

**Affiliations:** College of Basic Medicine and Forensic Medicine, Henan University of Science and Technology, Luoyang 471003, China; wjf20220222@163.com (J.W.);

**Keywords:** rigid planar structures, quinazoline, 1,2,3-triazole, DNA damage, HepG2 cells

## Abstract

Compounds with rigid planar structures can insert into tumor cell DNA, thereby inducing DNA damage in tumor cells. In this study, quinazoline, a compound with a planar structure, was used as the core scaffold. A rigid planar 1,2,3-triazole moiety was introduced into its structure, and its activity was tested on HepG2 liver cancer cells. The results showed that most compounds exhibited inhibitory effects on HepG2 cells, and the IC50 values of the most effective compounds were 3.08 ± 0.37 μM and 3.60 ± 0.53 μM. We found that the designed compounds significantly upregulated the expression of γ-H2AX in tumor cells, inducing DNA damage while reducing PARP levels, thereby weakening the DNA damage repair capacity of tumor cells and leading to apoptosis. Additionally, these compounds inhibited the migration and invasion of HepG2 cells. One of the compounds was found to be low in toxicity in mice, suggesting its potential as a targeted DNA anti-tumor drug.

## 1. Introduction

The occurrence of tumors severely impacts human health, as uncontrolled cell growth and tumor metastasis are major characteristics of cancer, making it one of the leading causes of death worldwide. Developing effective anti-tumor drugs has become a crucial focus of medical research. Among various anti-cancer drug development strategies, anti-tumor drugs that induce DNA damage have gained significant attention due to their unique mechanisms of action and notable therapeutic effects [1,2]. DNA is the genetic material of cells, and maintaining its stability is essential for cell survival. By specifically inducing DNA damage in tumor cells, proliferation can be effectively halted, and apoptosis can be triggered, thereby achieving an anti-tumor effect [3,4]. Traditional chemotherapy drugs, such as cisplatin, cyclophosphamide, and paclitaxel, induce DNA damage by binding to DNA or interfering with DNA replication, leading to tumor cell apoptosis. However, it has been found that when tumor cell DNA is damaged, a large number of DNA damage response factors (PARP) rapidly accumulate at the damage sites to coordinate the repair process. This allows for tumor cells to repair the DNA damage caused by anti-tumor drugs. Therefore, developing lead compounds that not only induce DNA damage in tumor cells but also inhibit their damage response and repair mechanisms is crucial for creating novel targeted DNA-damaging anti-tumor drugs [5,6,7,8,9].

**Figure 1:**
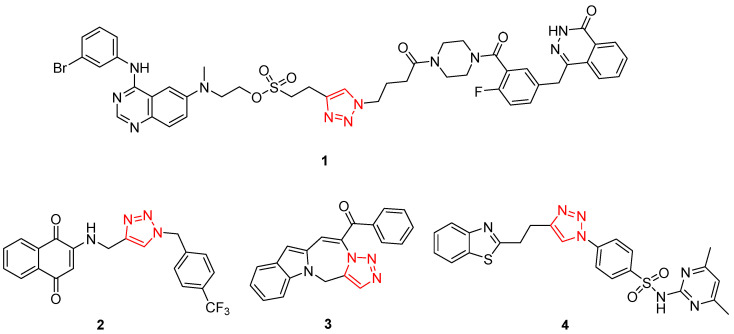
Structures of compound **1**, compound **2**, compound **3**, and compound **4** (the red is 1,2,3-Triazole structure).

1,2,3-Triazole is a five-membered heterocyclic compound with a rigid planar structure. It can interact with various enzymes and receptors in the body through non-covalent interactions, such as hydrophobic effects, van der Waals forces, and hydrogen bonds, exhibiting anti-tumor activity [10,11,12,13]. Additionally, it can exert anti-tumor effects by inserting into tumor cell DNA and inducing DNA damage. This makes it a hot topic in current research for developing anti-tumor drugs that induce DNA damage [14,15]. For instance, Jean-Claude designed a novel conjugate molecule, compound **1** (Figure 1), which integrates multiple functions (e.g., inhibiting EGFR phosphorylation, inducing DNA damage, and blocking DNA repair) into a single molecule. Compound **1** inhibits EGFR activity, damages DNA in a dose-dependent manner, and kills cancer cells by inhibiting DNA repair enzymes (PARP). In this molecule, gefitinib serves as an EGFR inhibitor, olaparib is used to block DNA repair, and 1,2,3-triazole functions as a DNA-damaging group. Compound **1** effectively eliminates the resistance observed when gefitinib and olaparib are used alone or in combination [16]. Khoshneviszadeh’s team synthesized novel aminonaphthoquinone–1,2,3-triazole hybrids based on the rigid planar aminonaphthoquinone structure, which induces DNA damage in tumor cells. They tested the compounds’ inhibitory activity on three tumor cell lines (MCF-7, HT-29, and MOLT-4) using an MTT method. The results showed that compound **2** (Figure 1) had inhibition rates with IC_50_ values of 10.4 μM, 6.8 μM, and 8.4 μM for these cell lines, respectively. Moreover, compound **2** caused cell cycle arrest at the G0/G1 phase in MCF-7 cells [17]. Nanduri’s group developed 1,2,3-triazole-fused indolo- and pyrrolo[1,4]diazepines with rigid planar structures. These compounds were tested for in vitro activity against various tumor cells, revealing that compound **3** (Figure 1) exhibited strong inhibitory activity against many cell lines. At a concentration of 2.5 μM, compound **3** completely inhibited colony formation in breast cancer cells (MDA-MB-468). Cell cycle analysis indicated that compound **3a** arrested the cell cycle in the G1 phase and had DNA-binding affinity, inducing apoptosis in tumor cells [18]. Aouad’s group synthesized a series of benzothiazole/isatin compounds linked to 1,2,3-triazole moieties and terminal sulfa drugs. They tested these compounds for EGFR enzyme inhibitory activity and in vitro tumor cell inhibition. Compound **4** (Figure 1) showed stronger EGFR inhibitory activity than erlotinib (IC_50_ of 104.29 nM). Molecular docking analysis confirmed that the 1,2,3-triazole ring could form hydrogen bonds with Met769, Gly772, and Leu694 in the EGFR receptor. Compound **4** effectively inhibited the growth of HepG2 cells, with an IC_50_ of 1.491 μM, causing cell cycle arrest in the G2/M phase and inducing DNA damage, leading to tumor cell apoptosis [19].

**Figure 2:**
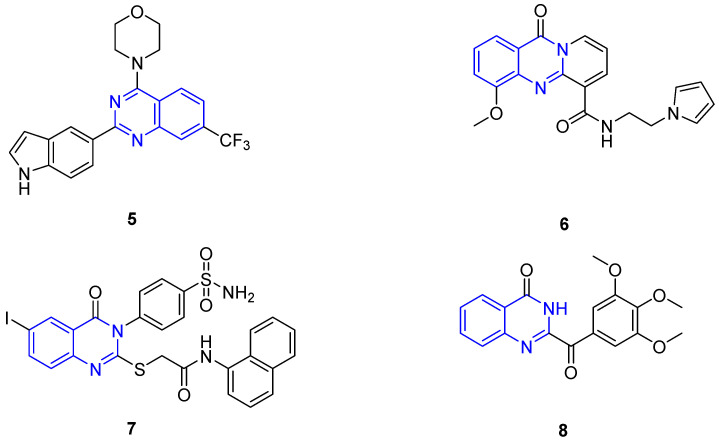
Structures of compound **5**, compound **6**, compound **7,** and compound **8** (the blue is quinazoline structure).

The quinazoline structure also possesses a rigid planar characteristic and is often utilized in the development of anti-tumor drugs due to its low toxicity and high biological activity. For example, the Yu research group designed compound **5** (Figure 2), which features a quinazoline structure and exhibits strong inhibitory activity against HepG2 cells. This compound can inhibit the migration of HepG2 cells in a dose- and time-dependent manner, promote apoptosis, and suppress the phosphorylation levels of AKT and mTOR in HepG2 cells [20]. Ranjbar’s group synthesized a new series of quinazolin-4(3H)-one-based compounds, among which compound **6** (Figure 2) demonstrated an inhibitory effect on HepG2 cells comparable to DOX (IC_50_ = 2.07 μM). It also showed strong affinity for tumor cell DNA and induced apoptosis in HepG2 cells [21]. Ghorab’s team synthesized a series of new quinazoline sulfonamide conjugates (2–16), with compound **7** (Figure 2) acting as a dual-target inhibitor of EGFR T790M (IC_50_ = 0.0728 μM) and VEGFR-2 (IC_50_ = 0.0523 μM), inducing apoptosis and G2/M phase cell cycle arrest in MCF-7 cells [22]. Gu’s group synthesized a series of 2-benzoylquinazolin-4(3H)-one derivatives, with compound **8** (Figure 2) exhibiting good inhibitory activity against HepG2 cells (IC_50_ = 1.22 μM). Compound **8** caused G2/M phase arrest in HepG2 cells and induced apoptosis by promoting the generation and upregulation of apoptosis-related proteins through reactive oxygen species [23,24].

Given that both the 1,2,3-triazole and quinazoline structures possess rigid planar characteristics and are widely used in the development of anti-tumor drugs, we propose, as part of our ongoing efforts to develop novel anti-tumor drugs, to modify the quinazoline structure with a 1,2,3-triazole group. We hope to discover lead compounds capable of inducing DNA damage in tumor cells while inhibiting DNA damage repair, which could be further developed into therapeutic agents for cancer treatment.

## 2. Chemistry

**Scheme 1:**
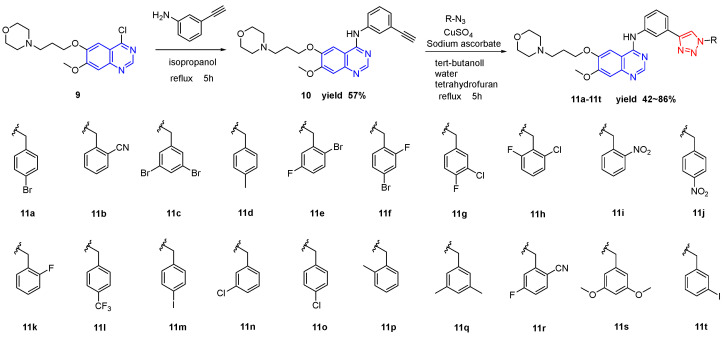
The reaction routes to gefitinib-conjugated 1,2,3-triazole derivatives (the blue is quinazoline structure, the red is 1,2,3-Triazole structure).

In this synthesis route, 4-(3-((4-chloro-7-methoxyquinazolin-6-yl)oxy)propyl)morpholine (compound **9**) underwent an acylation reaction with 3-aminophenylacetylene, yielding terminal alkyne compound **10**. These compounds were then reacted with various azide compounds, resulting in the formation of 20 novel target compounds (**11a**–**11t**), as shown in Scheme 1. The structures of the target compound were confirmed through ^1^H and ^13^C nuclear magnetic resonance (^1^H NMR and ^13^C NMR) spectroscopy.

## 3. Results and Discussion

### 3.1. Gefitinib Derivatives Inhibited the Cell Viability of HepG2 Cells

**Figure 3:**
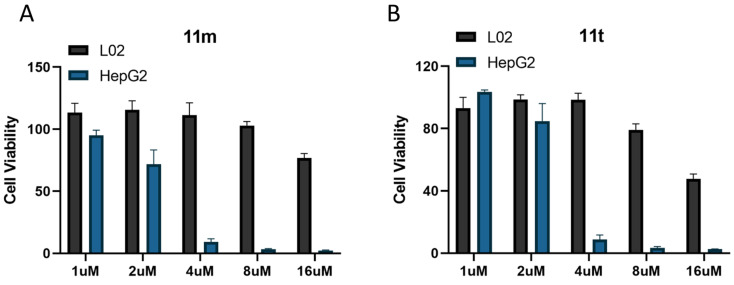
Evaluation of cell viability of L02 and HepG2 by compounds **11m** and **11t**. (**A**) Cell viability of HepG2 cells treated with 1, 2, 4, 8, or 16 μM of **11m** compounds. (**B**) Cell viability of HepG2 cells treated with 1, 2, 4, 8, or 16 μM of **11t** compounds.

To evaluate the potential cytotoxicity of the newly synthesized compounds **11a**–**11t**, we selected the HepG2 human tumor cell line and treated the cells with 10 μM of each compound, using gefitinib as a positive control. After 72 h of treatment, cell viability and cytotoxicity were assessed using a CCK8 assay kit. The results presented in Table 1 indicated that, at the 10 μM concentration, the compounds generally exhibited superior inhibitory activity against HepG2 cells compared to gefitinib. We further selected compounds with higher inhibition rates than gefitinib to investigate their half-maximal inhibitory concentration (IC_50_) against HepG2 cells. Cells were treated with various doses of these compounds for 72 h, and cell viability was measured. Based on these viability results, we calculated the inhibition rates and determined the IC_50_ values for each compound using GraphPad Prism software9.0. The findings, shown in Table 2, revealed that compounds **11m** and **11t** demonstrated particularly notable anticancer activity, with IC_50_ values of 3.08 ± 0.37 μM and 3.60 ± 0.53 μM, respectively (Table 2). Additionally, we assessed the toxicity of compounds **11m** and **11t** against normal liver cells (L02) and found that they exhibited a favorable safety profile (Figure 3).

### 3.2. Cell Apoptosis of Novel Synthetic Compounds

**Figure 4:**
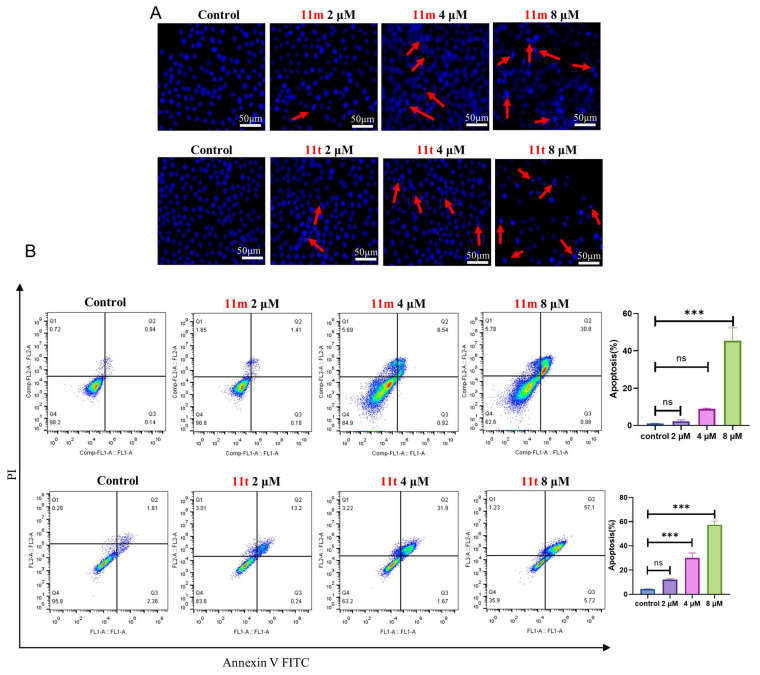
Cell apoptosis induced by **11m** and **11t** in HepG2 cells. (**A**) Cell apoptosis morphological changes detected by DAPI staining. (**B**) Apoptosis quantification detected by Annexin V-FITC/PI staining. Data were mean ± SD. *n* = 3 for each concentration. ns means *p* > 0.05, *** *p* < 0.001, vs. control group. (The red arrow indicates the abnormal nuclear morphology of apoptosis).

To verify whether the inhibition of cell proliferation and cytotoxic effects of these compounds are related to apoptosis, we selected compounds **11m** and **11t**, which showed good inhibitory effects on tumor cell proliferation, for further experiments. To confirm the apoptosis-inducing activity of **11m** and **11t** in liver cancer cells, HepG2 cells were treated with different concentrations of compounds **11m** and **11t** for 48 h, followed by DAPI staining. The cells were observed under a fluorescence microscope, and representative images are shown in Figure 4A. Compared to the control group, HepG2 cells treated with compounds **11m** and **11t** exhibited typical apoptotic characteristics, such as enhanced nuclear staining and even nuclear condensation and fragmentation, with increasing drug concentrations. The apoptosis-related results indicate that both **11m** and **11t** significantly promote apoptosis in human liver cancer HepG2 cells in a concentration-dependent manner.

HepG2 cells were treated with DMSO or different concentrations of **11m** and **11t** for 48 h, followed by staining with Annexin-V and PI. The proportion of apoptotic cells was then analyzed using flow cytometry. As shown in Figure 4B, the total proportion of apoptotic HepG2 cells after treatment with compound **11m** was 1.59% (2 μM), 9.46% (4 μM), and 31.66% (8 μM), while treatment with compound **11t** resulted in total apoptosis proportions of 13.44% (2 μM), 33.57% (4 μM), and 62.82% (8 μM). Compared to the control group, the proportion of apoptotic cells increased gradually with increasing drug concentrations (*p* < 0.01, *p* < 0.05). These results suggest that both **11m** and **11t** significantly promote apoptosis in the HepG2 liver cancer cell line in a concentration-dependent manner.

### 3.3. Compound ***11m*** and ***11t*** Affected Cell Growth

**Figure 5:**
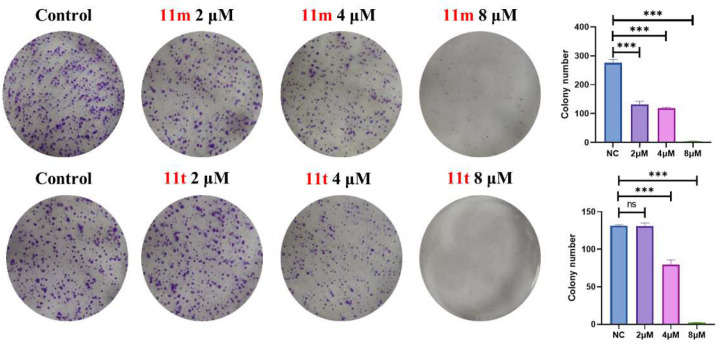
Colony formation pictures of the HepG2 treated with **11m** or **11t**. ns means *p* > 0.05, *** *p* < 0.001, vs. control group.

A colony formation assay is used to assess the ability of adherent cells to proliferate and form cell colonies on a plate, making it one of the key methods for evaluating cell proliferation. We also employed the colony formation assay to further confirm that compounds **11m** and **11t** can inhibit liver cancer cell proliferation (as shown in Figure 5). HepG2 cells were treated with low, medium, and high concentrations (2, 4, and 8 μmol/L) of the compounds for 7 days, followed by counting the number of cell colonies and analyzing the proliferation inhibition rate. The results showed that the number of cell colonies in the treatment groups was significantly reduced in a concentration-dependent manner. At a concentration of 4 μM, the compounds began to noticeably inhibit colony formation in HepG2 cells, and at 8 μM, colony formation was almost completely suppressed, indicating that compounds **11m** and **11t** exert a strong antiproliferative effect on HepG2 cells.

### 3.4. Compound ***11m*** and ***11t*** on the Migration of HepG2 Cells

**Figure 6:**
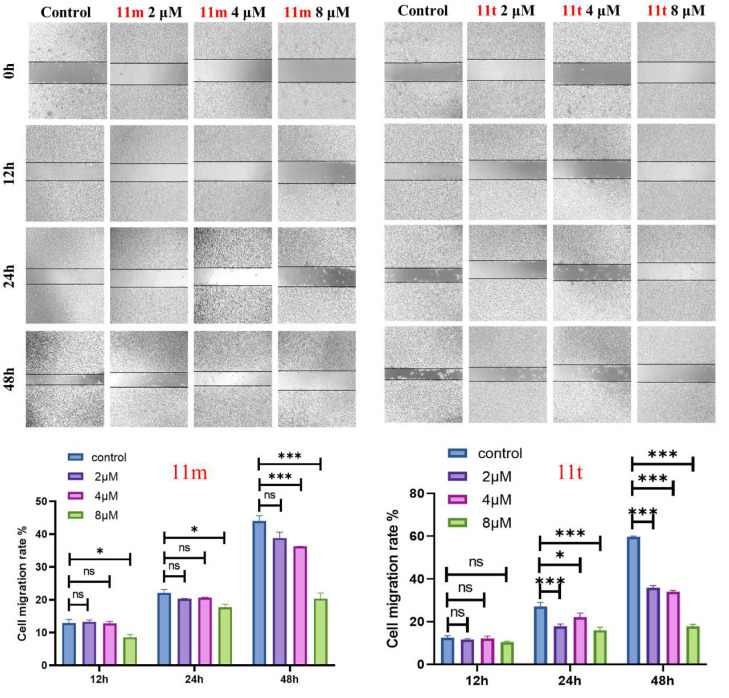
Cell migration inhibition induced by **11m** and **11t** in HepG2 cells. In vitro cell scratch assays. Photographs of cells at 0, 12, 24, and 48 h after treatment with different concentrations of drugs. Data were mean ± SD. *n* = 3 for each concentration. ns means *p* > 0.05, * *p* < 0.05, *** *p* < 0.001, vs. control group.

**Figure 7:**
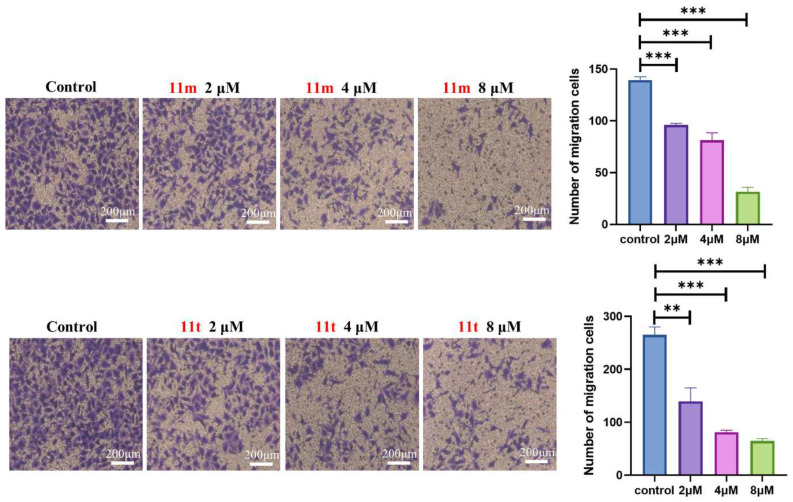
Cell migration inhibition induced by **11m** and **11t** in HepG2 cells. Transwell migration assays. The migration of cells to the lower surface of the chamber was determined by crystal violet staining. Data were mean ± SD. *n* = 3 for each concentration. ** *p* < 0.01, *** *p* < 0.001, vs. control group.

To investigate the effect of the compounds on the migration of wild-type liver cancer cells, we conducted wound healing and Transwell assays to assess changes in the migration ability of HepG2 cells after treatment with compounds **11m** and **11t**. The results of the wound-healing assay are shown in Figure 6, indicating that after treatment with compound **11m**, the wound-healing rates of HepG2 cells at 12, 24, and 48 h were 14.32%, 20.33%, and 41.13% (2 μM); 13.89%, 20.60%, and 36.35% (4 μM); and 8.96%, 16.39%, and 18.48% (8 μM), respectively. After treatment with compound **11t**, the corresponding wound-healing rates were 11.03%, 16.02%, and 35.36% (2 μM); 14.18%, 25.67%, and 33.66% (4 μM); and 9.87%, 13.26%, and 19.54% (8 μM). The wound-healing rates in the compound-treated groups were significantly lower than those in the control group, and this reduction was concentration-dependent (*p* < 0.01, *p* < 0.05). The Transwell migration assay, another important method for evaluating cell migration, involves seeding tumor cells in a chamber and assessing their migration ability by counting the number of cells that pass through the membrane. The Transwell migration assay results also demonstrated that, compared to the control group, the number of cells that migrated through the microporous membrane to the lower surface of the chamber was significantly reduced in the compound-treated groups (*p* < 0.01) (Figure 7). These findings indicate that compounds **11m** and **11t** can inhibit the migration ability of liver cancer cells in a concentration-dependent manner.

### 3.5. Compound ***11m*** and ***11t*** Stimulated DNA Damage of Tumor Cells

**Figure 8:**
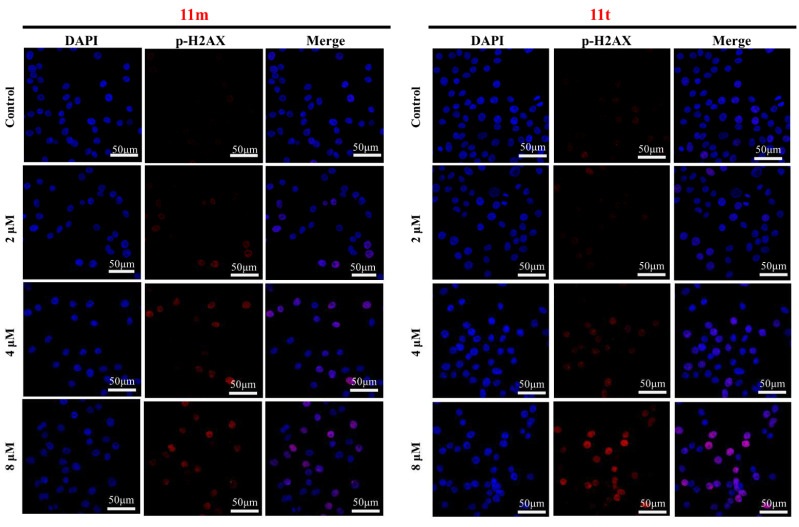
DNA damage of HepG2 cells induced by **11m** and **11t**. The expression of DNA damage marker p-H2ax in HepG2 cells induced by **11m** and **11t** was observed using immunofluorescence.

Previous studies have reported that the phosphorylation of H2AX is a highly specific and sensitive molecular marker of DNA damage. To determine whether the inhibitory effects of compounds **11m** and **11t** on HepG2 cells are related to p-H2AX, we conducted immunofluorescence and Western blot analyses. The results showed that both **11m** and **11t** increased the expression of p-H2AX in HepG2 cells, indicating that the inhibitory effects and apoptosis induction by these compounds are associated with DNA damage (Figure 8).

### 3.6. Assessment of Apoptosis Induced by Compounds ***11m*** and ***11t***

**Figure 9:**
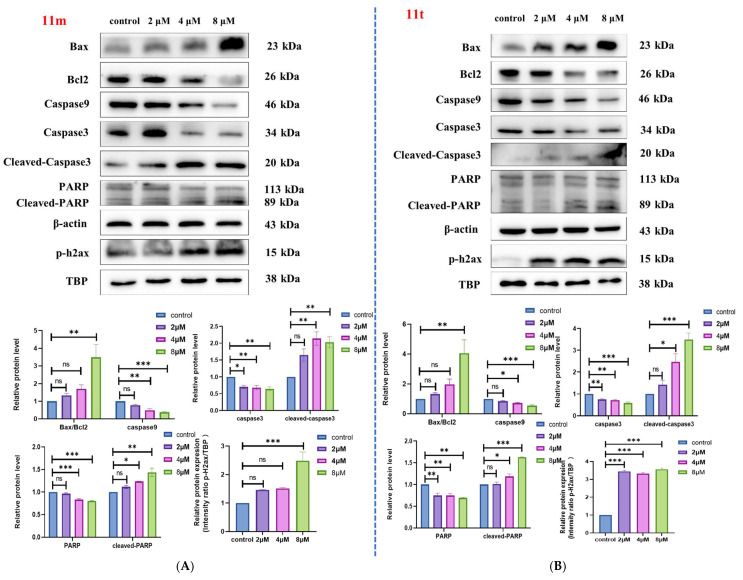
Assessment of apoptosis induced by compounds **11m** and **11t**. (**A**) Western-blotting results of the protein levels in HepG2 cells treated with 2, 4, or 8 μM of **11m** compounds. (**B**) Western-blotting results of the protein levels in HepG2 cells treated with 2, 4, or 8 μM of **11t** compounds. (ns means *p* > 0.05, * *p* < 0.05, ** *p* < 0.01, *** *p* < 0.001, vs. control group).

We conducted an in-depth study on the mechanisms of apoptosis induced by compounds **11m** and **11t** (Figure 9). The Bcl-2 protein family is located upstream in the apoptosis signaling pathway, with Bcl-2 exerting anti-apoptotic and pro-proliferative effects by inhibiting the release of cytochrome C (cyt-c) from the mitochondria. Previous studies have reported that many anticancer drugs exert pro-apoptotic effects by downregulating Bcl-2. When apoptosis occurs, the expression level of Bcl-2 is suppressed, cyt-c is released, and it binds to apoptotic protease activating factor (Apaf-1) to form the apoptosome, triggering a cascade reaction involving the Caspase family proteins. The apoptosome first promotes the activation of Caspase-9, which in turn activates Caspase-3, forming cleaved Caspase-3. Cleaved Caspase-3 further activates downstream PARP proteins, ultimately promoting apoptosis. We examined the expression levels of Bcl-2, Bax, Caspase-9, Caspase-3, and PARP proteins and found that in the groups treated with compounds **3m** and **3t**, the expression levels of Bcl-2, Caspase-9, Caspase-3, and PARP decreased, while the levels of cleaved Caspase-3 and cleaved PARP increased. These results suggest that compounds **11m** and **11t** may effectively induce apoptosis in liver cancer cells through the Bcl-2/Caspase-3/PARP signaling pathway. After treating HepG2 cells with different concentrations of the compounds for 48 h, we extracted cellular proteins and used Western blotting to detect changes in the expression of apoptosis-related proteins. The results showed that the expression of the apoptosis-related protein Bcl-2 decreased with increasing drug concentration, while the expression of Bax increased. The expression levels of Caspase-3, Caspase-9, and PARP decreased with increasing drug concentration, whereas the levels of cleaved Caspase-3 and cleaved PARP increased. This indicates that apoptosis was significantly stronger in the high-concentration drug groups compared to the low-concentration groups and the control group (*p* < 0.01, *p* < 0.05).

### 3.7. The Effect of Compound ***11t*** on Liver Injury in Mice

**Figure 10:**
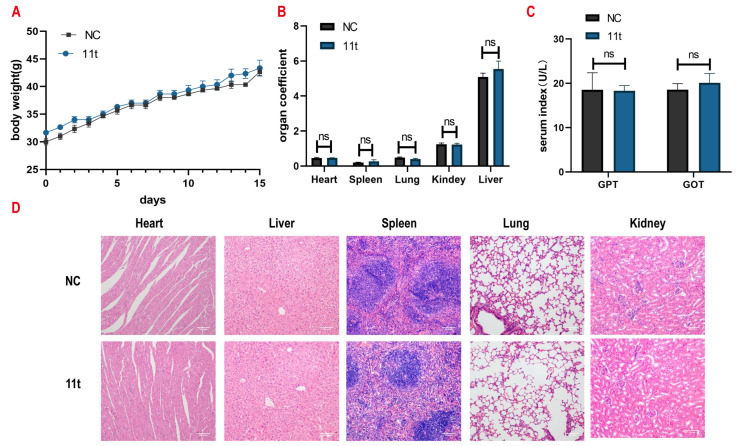
Compound **11t** in vivo toxicity of mice. (**A**) Acute toxicity experiments of compound **11t** were conducted in mice. Mouse body weight changed. (**B**) The acute toxicity experiments examined the effects of compound **11t** on mouse organs (**C**) Effect of acute toxicity experimental studies on blood biochemical indices in mice. (**D**) H&E staining was performed on various organs of mice treated with compound **11t**. (ns means *p* > 0.05, vs. NC group).

To further assess the safety of the compounds, we conducted an acute toxicity experiment on mice. As shown in Figure 10A, from the first gavage to the continuous observation on day 14, there were no statistically significant differences in body weight among the groups compared to the control group (*p* > 0.05). No pathological morphology was observed during organ morphological examinations, and there were no statistically significant differences in the organ indices of the heart, liver, spleen, lungs, and kidneys compared to the control group (Figure 10B).

Liver function was evaluated by measuring serum glutamic pyruvic transaminase (GPT) and glutamic oxaloacetic transaminase (GOT) levels (Figure 10C). GPT and GOT are commonly used indicators for assessing liver function, reflecting physiological and pathological changes in the liver. Elevated serum levels of GPT and GOT indicate damage to liver cells or mitochondria. There were no statistically significant differences in biochemical indicators between the treatment and control groups (*p* > 0.05), and all values were within the normal range. Hematoxylin and eosin staining was used for histological analysis of the hearts, livers, spleens, lungs, and kidneys of the mice, further confirming that there were no significant toxic characteristics in the organs at this drug concentration (Figure 10D). This provides additional evidence for the high safety of the compound and experimental data for clinical drug safety (*p* > 0.05).

## 4. Conclusions

Based on the mechanism that compounds with a rigid planar structure can intercalate into tumor cell DNA, thereby inducing DNA damage in tumor cells, this study used quinazoline with a planar structure as the core scaffold and introduced the rigid planar structure of 1,2,3-triazole into it. Activity tests on HepG2 liver cancer cells revealed that the **11m** and **11t** series of compounds exhibited inhibitory effects on HepG2 cells, with IC_50_ values of 3.08 ± 0.37 μM and 3.60 ± 0.53 μM, respectively. Both compounds significantly increased the expression levels of γ-H2AX in the tumor cells, induced DNA damage, reduced PARP levels, and impaired the DNA damage repair process in the tumor cells, ultimately leading to apoptosis. Additionally, these compounds inhibited the migration and invasion of HepG2 cells. Among them, compound **11t** exhibited low toxicity in mice, showing potential as a promising DNA-targeted antitumor agent.

## 5. Experimental

### 5.1. Materials and Chemistry

Gefitinib derivatives were synthesized in-house. Dulbecco’s modified Eagle medium (DMEM) was obtained from LifeTech (Grand Island, NY, USA). Fetal bovine serum (FBS) was purchased from Gibco (Grand Island, NY, USA). NE-PER™ Nuclear and Cytoplasmic Extraction Reagents and BCA protein assay kit were purchased from Thermo Fisher Scientific (Rockford, IL, USA). Griess reagent, β-actin antibody, and Protease Inhibitor Cocktail were obtained from Sigma-Aldrich (St. Louis, MO, USA). Caspase 3 antibody, Caspase 9 antibody, PARP antibody, bax antibody, and bcl-2 antibody were purchased from Cell Signaling Technology (Beverly, MA, USA).

#### Synthesis of Gefitinib Derivative **11a** (Compounds **11b**–**11t**, the Method Are Applicable to the Preparation of Compound **11a**)

In a reaction flask, add 4-(3-((4-chloro-7-methoxyquinazolin-6-yl)oxy)propyl)morpholine (0.1 mol) to 500 mL of isopropanol; then, add meta-aminophenylacetylene (0.11 mol). Heat the mixture to reflux and maintain the reaction for 5 h, during which a large amount of solid will form. Monitor the reaction by TLC until the starting materials are completely consumed. After cooling the mixture to 0 °C and stirring for 30 min, filter the mixture and dry it to obtain compound **2**.

In a new reaction flask, sequentially add compound **2** (0.4 g), 4-bromobenzyl azide (0.4 g), and a mixed solution of *tert*-butanol, water, and tetrahydrofuran (30 mL). Then, add copper(II) sulfate pentahydrate (0.2 g) and sodium ascorbate (0.4 g). Slowly heat the mixture to 70 °C and react for 5 h. After the reaction, add 20 mL of dichloromethane, stir, and then filter the reaction mixture. Extract the aqueous layer with dichloromethane twice, combine the organic phases, dry over anhydrous magnesium sulfate, and concentrate. Recrystallize the residue from a certain amount of anhydrous methanol to obtain compound **11a**.

The spectroscopic characterization of compounds **11a**–**11t** is provided as follows:

N-(3-(1-(4-bromobenzyl)-1H-1,2,3-triazol-4-yl)phenyl)-7-methoxy-6-(3-morpholinopropoxy)quinazolin-4-amine (compound **11a**): HR MS (ESI) *m*/*z*: calcd for C_31_H_32_BrN_7_O_3_ [M+H]^+^ 630.1823, found 630.1819. Yellow solid, yield 71%, Purity 97.7%; m.p. 177–178 °C, ^1^H NMR (400 MHz, DMSO-d6) δ 9.59 (s, 1H), 8.65 (s, 1H), 8.25 (d, J = 4.0 Hz, 1H), 7.94–7.88 (m, 2H), 7.62–7.60 (m, 2H), 7.55 (d, J = 8.0 Hz, 1H), 7.45 (t, J = 8.0 Hz, 1H), 7.34 (d, J = 8.0 Hz, 2H), 5.66 (s, 2H), 4.21 (t, J = 4.0 Hz, 2H), 3.95 (s, 3H), 3.59 (t, J = 4.0 Hz, 4H), 2.04–1.97 (m, 3H), 1.23 (s, 3H). ^13^C NMR (100 MHz, DMSO-d6) δ 156.73, 154.79, 148.75, 147.16, 140.54, 135.86, 132.22, 131.30, 130.70, 129.50, 122.41, 122.17, 121.94, 120.81, 119.33, 103.28, 67.60, 66.56, 56.34, 55.42, 53.83, 52.78, 31.75, 29.47, 29.04, 27.01, 26.24, 22.55.

2-((4-(3-((7-methoxy-6-(3-morpholinopropoxy)quinazolin-4-yl)amino)phenyl)-1H-1,2,3-triazol-1-yl)methyl)benzonitrile (Compound **11b**): HR MS (ESI) *m*/*z*: calcd for C_32_H_32_N_8_O_3_ [M+H]^+^ 577.2670, found 577.2671. Yellow solid, yield 67%, Purity 95.7%; m.p. 167–168 °C, ^1^H NMR (400 MHz, DMSO-d6) δ 9.60 (s, 1H), 8.69 (s, 1H), 8.49 (s, 1H), 8.27 (s, 1H), 7.96–7.89 (m, 3H), 7.76 (t, J = 8.0 Hz, 1H), 7.61–7.56 (m, 2H), 7.49–7.45 (m, 2H), 7.22 (s, 1H), 5.89 (s, 2H), 4.21 (t, J = 8 Hz, 2H), 3.95 (s, 3H), 3.60 (t, J = 8.0 Hz, 4H), 2.43 (m, 4H), 2.05–2.01 (m, 2H). ^13^C NMR (100 MHz, DMSO-d6) δ 156.79, 154.83, 148.73, 147.07, 140.56, 139.17, 134.37, 133.91, 131.20, 129.74, 122.61, 122.48, 120.85, 119.36, 117.47, 111.73, 103.25, 67.60, 66.56, 56.33, 55.42, 53.84, 51.73, 26.23.

N-(3-(1-(3,5-dibromobenzyl)-1H-1,2,3-triazol-4-yl)phenyl)-7-methoxy-6-(3-morpholinopropoxy)quinazolin-4-amine (compound **11c**): HR MS (ESI) *m*/*z*: calcd for C_31_H_31_Br_2_N_7_O_3_ [M+H]^+^ 710.0907, found 710.0906. Yellow solid, yield 75%, Purity 96.6%; m.p. 179–180 °C, ^1^H NMR (400 MHz, DMSO-d6) δ 9.61 (s, 1H), 8.71 (s, 1H), 8.27 (s, 1H), 8.06–7.86 (m, 3H), 7.64–7.44 (m, 5H), 5.69 (s, 2H), 4.21 (t, J = 8.0 Hz, 2H), 3.97 (s, 3H), 3.59 (t, J = 4.0 Hz, 5H), 2.51–2.49 (m, 5H), 2.03–1.99 (m, 2H). ^13^C NMR (100 MHz, DMSO-d6) δ 148.83, 147.21, 140.81, 140.57, 133.73, 131.21, 130.66, 129.52, 123.22, 122.48, 122.41, 120.84, 119.37, 67.61, 66.59, 56.36, 55.42, 53.86, 52.02, 26.29.

7-methoxy-N-(3-(1-(4-methylbenzyl)-1H-1,2,3-triazol-4-yl)phenyl)-6-(3-morpholinopropoxy)quinazolin-4-amine (compound **11d**): HR MS (ESI) *m*/*z*: calcd for C_32_H_35_N_7_O_3_ [M+H]^+^ 566.2874, found 566.2873. Yellow solid, yield 62%, Purity 96.5%; m.p. 168–169 °C, ^1^H NMR (400 MHz, DMSO-d6) δ 9.60 (s, 1H), 8.62 (s, 1H), 8.25 (s, 1H), 8.17–7.68 (m, 3H), 7.55 (d, J = 4.0 Hz, 1H), 7.44 (t, J = 8 Hz, 1H), 7.29–7.19 (m, 5H), 5.60 (s, 2H), 4.22–4.19 (m, 3H), 3.98 (s, 3H), 3.59 (m, 4H), 2.50 (m, 4H), 2.31–2.39 (m, 4H), 2.32–2.30 (m, 1H), 2.03–2.01 (m, 2H). ^13^C NMR (100 MHz, DMSO-d6) δ 167.44, 154.87, 147.08, 140.53, 138.01, 133.47, 132.20, 132.00, 131.42, 129.81, 129.73, 129.49, 129.13, 129.97, 128.47, 128.41, 122.37, 121.96, 120.79, 119.30, 67.60, 66.57, 65.49, 56.37, 55.41, 53.83, 53.34, 30.47, 26.26, 21.17, 19.11, 13.96.

N-(3-(1-(2-bromo-5-fluorobenzyl)-1H-1,2,3-triazol-4-yl)phenyl)-7-methoxy-6-(3-morpholinopropoxy)quinazolin-4-amine (compound **11e**): HR MS (ESI) *m*/*z*: calcd for C_37_H_35_N_9_O_7_ [M+H^+^]:718.2732, found 728.2731. Yellow solid, yield 65%, Mp: 165–166 °C. ^1^H NMR (400 MHz, DMSO-d6) δ 9.63 (s, 1H), 8.64 (s, 1H), 8.26 (s, 1H), 8.13–7.89 (m, 2H), 7.76 (s, 1H), 7.59 (s, 1H), 7.45–7.18 (m, 4H), 5.75 (s, 2H), 4.57–4.23 (m, 3H), 3.98 (s, 3H), 3.59 (m, 4H), 2.51 (m, 5H), 2.03–2.01 (m, 2H). ^13^C NMR (100 MHz, DMSO-d6) δ 160.70, 154.65, 148.96, 140.53, 137.44, 137.36, 135.24, 135.16, 131.25, 129.50, 122.56, 120.91, 119.42, 118.42, 118.18, 117.97, 67.62, 66.63, 56.39, 55.44, 53.89, 53.38, 26.32.

N-(3-(1-(4-bromo-2-fluorobenzyl)-1H-1,2,3-triazol-4-yl)phenyl)-7-methoxy-6-(3-morpholinopropoxy)quinazolin-4-amine (compound **11f**): HR MS (ESI) *m*/*z*: calcd for C_37_H_35_N_9_O_7_ [M+H^+^]: 718.2732, found 728.2731. Yellow solid, yield 81%, Mp: 169–170 °C. ^1^H NMR (400 MHz, DMSO-d6) δ 9.60 (s, 1H), 8.64 (s, 1H), 8.26 (s, 1H), 8.02–7.90 (m, 2H), 7.65 (d, J = 8.0 Hz, 1H), 7.57–7.36 (m, 4H), 5.71 (s, 2H), 4.38–4.23 (m, 3H), 3.97 (s, 3H), 3.67–3.36 (m, 5H), 2.51 (m, 5H), 2.03–1.99 (m, 2H). ^13^C NMR (100 MHz, DMSO) δ 161.79, 159.29, 154.77, 148.80, 147.05, 140.55, 132.86, 132.82, 131.24, 129.48, 128.59, 128.56, 122.92, 122.76, 122.66, 122.46, 122.25, 120.83, 119.74, 119.49, 119.34, 103.38, 67.62, 66.62, 56.35, 55.44, 53.88, 47.19, 47.16, 26.31.

N-(3-(1-(3-chloro-4-fluorobenzyl)-1H-1,2,3-triazol-4-yl)phenyl)-7-methoxy-6-(3-morpholinopropoxy)quinazolin-4-amine (compound **11g**): HR MS (ESI) *m*/*z*: calcd for C_31_H_31_ClFN_7_O_3_ [M+H^+^]: 605.2234, found 605.2234. Yellow solid, yield 79%, Mp: 179–181 °C. ^1^H NMR (400 MHz, DMSO-d6) δ 9.60 (s, 1H), 8.61 (s, 1H), 8.25 (s, 1H), 8.06–7.90 (m, 3H), 7.57–7.29 (m, 6H), 5.71 (s, 2H), 4.25–4.15 (m, 3H), 3.97 (s, 3H), 3.65–3.59 (m, 5H), 2.51 (m, 5H), 2.02–1.99 (m, 2H). ^13^C NMR (100 MHz, DMSO) δ 161.13, 154.75, 148.81, 146.92, 140.52, 133.07, 132.98, 131.27, 130.09, 129.48, 122.50, 122.36, 120.89, 119.39, 117.68, 117.43, 115.56, 115.35, 67.62, 66.61, 56.36, 55.43, 53.87, 50.72, 26.30.

N-(3-(1-(2-chloro-6-fluorobenzyl)-1H-1,2,3-triazol-4-yl)phenyl)-7-methoxy-6-(3-morpholinopropoxy)quinazolin-4-amine (compound **11h**): HR MS (ESI) *m*/*z*: calcd for C_31_H_31_ClFN_7_O_3_ [M+H^+^]: 604.2234, found 605.2232. Yellow solid, yield 75%, Mp: 177–180 °C. ^1^H NMR (400 MHz, DMSO-d6) δ 9.62 (s, 1H), 8.62 (s, 1H), 8.23 (s, 1H), 8.12–7.89 (m, 2H), 7.60–7.35 (m, 6H), 5.78 (s, 2H), 4.33–4.19 (m, 3H), 3.98 (s, 3H), 3.61–3.58 (m, 5H), 2.54–2.51 (m, 5H), 2.05–2.00 (m, 2H). ^13^C NMR (100 MHz, DMSO) δ 163.11, 160.62, 148.86, 146.69, 140.48, 135.50, 135.45, 132.39, 132.29, 131.25, 129.45, 129.13, 126.39, 126.36, 122.57, 122.32, 120.96, 115.60, 115.38, 67.60, 66.56, 56.38, 55.41, 53.83, 45.09, 30.47, 26.25.

7-methoxy-6-(3-morpholinopropoxy)-N-(3-(1-(2-nitrobenzyl)-1H-1,2,3-triazol-4-yl)phenyl)quinazolin-4-amine (compound **11i**): HR MS (ESI) *m*/*z*: calcd for C_31_H_33_N_8_O_5_ [M+H]^+^ 597.2568, found 597.2567. Yellow solid, yield 71%. ^1^H NMR (400 MHz, DMSO-d6) δ 9.62 (s, 1H), 8.64 (s, 1H), 8.28 (s, 1H), 8.18 (d, J = 8.0 Hz, 1H), 8.04–7.90 (m, 1H), 7.78 (t, J = 8.0 Hz, 1H), 7.66 (t, J = 8.0 Hz, 1H), 7.58 (d, J = 8.0 Hz, 1H), 7.47 (t, J = 4.0 Hz, 1H), 7.19 (t, J = 8.0 Hz, 1H), 5.76 (s, 2H), 4.21–3.97 (m, 5H), 3.60–3.51 (m, 5H), 2.51–2.49 (m, 5H), 2.04–1.97 (m, 2H). ^13^C NMR (100 MHz, DMSO) δ 148.83, 148.08, 147.03, 140.57, 134.93, 131.23, 131.18, 130.75, 130.20, 129.52, 125.52, 122.62, 122.87, 122.48, 120.85, 119.37, 103.44, 67.62, 66.61, 56.36, 55.43, 55.37, 53.87, 50.69, 26.30.

7-methoxy-6-(3-morpholinopropoxy)-N-(3-(1-(4-nitrobenzyl)-1H-1,2,3-triazol-4-yl)phenyl)quinazolin-4-amine (compound **11j**): HR MS (ESI) *m*/*z*: calcd for C_31_H_33_N_8_O_5_ [M+H^+^]: 597.2568, found 597.2567. Yellow solid, yield 55%, Mp: 171–173 °C. ^1^H NMR (400 MHz, DMSO-d6) δ 9.63 (s, 1H), 8.72 (s, 1H), 8.27 (d, J = 8.0 Hz, 3H), 7.91 (t, J = 12.0 Hz, 2H), 7.61–7.27 (m, 6H), 5.76 (s, 2H), 4.70 (s, 1H), 4.31–4.21 (m, 3H), 3.95 (s, 3H), 3.63–3.38 (m, 5H), 2.57–2.51 (m, 5H), 2.03–2.00 (m, 2H). ^13^C NMR (100 MHz, DMSO-d6) δ 156.80, 154.86, 148.77, 147.75, 147.25, 143.86, 140.49, 135.94, 131.91, 131.45, 131.22, 129.52, 129.35, 124.44, 122.55, 120.50, 120.89, 119.41, 103.24, 67.61, 66.55, 56.34, 55.41, 53.83, 52.63, 49.38, 26.24.

N-(3-(1-(2-fluorobenzyl)-1H-1,2,3-triazol-4-yl)phenyl)-7-methoxy-6-(3-morpholinopropoxy)quinazolin-4-amine (compound **11k**): HR MS (ESI) *m*/*z*: calcd for C_31_H_32_FN_7_O_3_ [M+H^+^]: 570.2623, found 570.2635. Yellow solid, yield 50%, Mp: 184–186 °C. ^1^H NMR (400 MHz, DMSO-d6) δ 9.61 (s, 1H), 8.64 (s, 1H), 8.25 (s, 2H), 7.91 (d, J = 8.0 Hz, 1H), 7.57 (d, J = 8.0 Hz, 1H), 7.47–7.40 (m, 3H), 7.29–7.25 (m, 2H), 5.73 (s, 2H), 4.36–4.20 (m, 3H), 3.99 (s, 3H), 3.61–3.59 (m, 5H), 2.54–2.51 (m, 5H), 2.04–2.00 (m, 2H). ^13^C NMR (100 MHz, DMSO-d6) δ 161.83, 159.37, 154.61, 148.88, 147.00, 140.54, 131.31, 131.25, 123.33, 123.18, 122.44, 122.23, 120.84, 119.33, 116.25, 116.04, 67.60, 66.53, 56.40, 55.41, 53.81, 47.60, 47.56, 26.23.

7-methoxy-6-(3-morpholinopropoxy)-N-(3-(1-(4-(trifluoromethyl)benzyl)-1H-1,2,3-triazol-4-yl)phenyl)quinazolin-4-amine (compound **11l**): HR MS (ESI) *m*/*z*: calcd for C_32_H_32_F_3_N_7_O_3_ [M+H^+^]: 620.2591, found 620.2604. ^1^H NMR (400 MHz, DMSO-d6) δ 9.61 (s, 1H), 8.71 (s, 1H), 8.28 (s, 1H), 8.03–7.91 (m, 2H), 7.78 (d, J = 8.0 Hz, 1H), 7.57 (d, J = 8.0 Hz, 3H), 7.47 (d, J = 8.0 Hz, 1H), 5.80 (s, 2H), 4.22–4.20 (m, 2H), 3.97 (s, 3H), 3.64–3.53 (m, 5H), 2.54–2.51 (m, 5H), 2.02–1.99 (m, 2H). ^13^C NMR (100 MHz, DMSO-d6) δ 154.72, 148.82, 147.23, 141.13, 140.57, 131.28, 129.51, 129.12, 126.24, 126.20, 122.43, 120.82, 119.35, 67.62, 66.62, 56.35, 55.44, 53.88, 52.86, 26.31.

N-(3-(1-(4-iodobenzyl)-1H-1,2,3-triazol-4-yl)phenyl)-7-methoxy-6-(3-morpholinopropoxy)quinazolin-4-amine (compound **11m**): HR MS (ESI) *m*/*z*: calcd for C_31_H_32_IN_7_O_3_ [M+H^+^]: 678.1690, found 678.2441. Yellow solid, yield 65%, Mp: 185–186 °C. ^1^H NMR (400 MHz, DMSO-d6) δ 9.59 (s, 1H), 8.65 (s, 1H), 8.26 (s, 1H), 7.97–7.89 (m, 2H), 7.77 (s, 2H), 7.55–7.43 (m, 2H), 7.18 (d, J = 8.0 Hz, 2H), 5.63 (s, 2H), 4.31–4.22 (m, 2H), 3.96 (s, 3H), 3.64–3.57 (m, 5H), 2.73–2.57 (m, 5H), 2.05–2.01 (m, 2H). ^13^C NMR (100 MHz, DMSO-d6) δ 156.74, 154.79, 148.69, 147.15, 140.54, 138.07, 136.23. 131.32, 130.72, 129.48, 122.38, 122.16, 120.80, 119.30, 103.32, 94.97, 67.54, 66.34, 56.34, 55.35, 53.67, 52.93, 31.77, 26.02.

N-(3-(1-(3-chlorobenzyl)-1H-1,2,3-triazol-4-yl)phenyl)-7-methoxy-6-(3-morpholinopropoxy)quinazolin-4-amine (compound **11n**): HR MS (ESI) *m*/*z*: calcd for C_31_H_32_ClN_7_O_3_ [M+H^+^]: 586.3005, found 586.3010. Yellow solid, yield 67%, Mp: 164–165 °C. ^1^H NMR (400 MHz, DMSO-d6) δ 9.60 (s, 1H), 8.70 (s, 1H), 8.27 (s, 1H), 7.99–7.90 (m, 2H), 7.57 (d, J = 4.0 Hz, 1H), 7.48–7.43 (m, 5H), 7.34 (s, 1H), 5.69 (s, 2H), 4.49 (s, 1H), 4.23–4.21 (m, 2H), 3.96 (s, 3H), 3.60–3.58 (m, 5H), 2.57–2.49 (m, 5H), 2.03 –1.99 (m, 2H). ^13^C NMR (100 MHz, DMSO-d6) δ 155.45, 154.76, 148.79, 147.19, 140.56, 138.83, 138.66, 133.81, 131.29, 131.24, 129.50, 128.67, 128.53, 128.37, 127.19, 122.42, 122.27, 120.81, 119.34, 103.36, 67.61, 66.61, 56.34, 55.44, 53.88, 53.17, 52.75, 26.31.

N-(3-(1-(4-chlorobenzyl)-1H-1,2,3-triazol-4-yl)phenyl)-7-methoxy-6-(3-morpholinopropoxy)quinazolin-4-amine (compound **11o**): HR MS (ESI) *m*/*z*: calcd for C_31_H_32_ClN_7_O_3_ [M+H^+^]: 586.3045, found 586.3041. Yellow solid, yield 70%, Mp: 165–167 °C. ^1^H NMR (400 MHz, DMSO-d6) δ 9.60 (s, 1H), 8.66 (s, 1H), 8.26 (s, 1H), 8.06–7.89 (m, 2H), 7.57–7.39 (m,7H), 5.68 (s, 2H), 4.22–4.21 (m, 2H), 3.96 (s, 3H), 3.68–3.59 (m, 5H), 2.58–2.41 (m, 5H), 2.02–1.99 (m, 2H). ^13^C NMR (100 MHz, DMSO-d6) δ 156.57, 154.72, 148.80, 147.16, 140.54, 135.45, 133.38, 131.32, 130.39, 129.49, 129.29, 122.41, 122.16, 120.81, 119.33, 103.40, 67.61, 66.60, 56.35, 55.43, 53.86, 52.73, 26.29.

7-methoxy-N-(3-(1-(2-methylbenzyl)-1H-1,2,3-triazol-4-yl)phenyl)-6-(3-morpholinopropoxy)quinazolin-4-amine (compound **11p**): HR MS (ESI) *m*/*z*: calcd for C_32_H_35_N_7_O_3_ [M+H^+^]: 566.3589, found 566.3591. Yellow solid, yield 65%, Mp: 169–171 °C. ^1^H NMR (400 MHz, DMSO-d6) δ 9.60 (s, 1H), 8.56 (s, 1H), 8.25 (s, 1H), 8.07–7.89 (m, 2H), 7.57 (s,1H), 7.44 (d, J = 8.0 Hz, 1H), 7.27–7.15 (m, 4H), 5.67 (s, 2H), 4.22–4.21 (m, 2H), 4.05–3.97 (m, 3H), 3.97 (s, 3H), 3.65–3.59 (m, 5H), 2.51–2.40 (m, 5H), 2.36 (s, 3H), 2.01 –1.99 (m, 2H). ^13^C NMR (100 MHz, DMSO-d6) δ 154.70, 148.84, 146.98, 140.53, 136.81, 134.56, 131.39, 130.93, 129.18, 128.83, 126.79, 122.41, 122.12, 120.83, 119.35, 67.62, 66.65, 60.23, 56.37, 55.45, 53.90, 51.65, 26.34, 21.23, 19.17, 14.55.

N-(3-(1-(3,5-dimethylbenzyl)-1H-1,2,3-triazol-4-yl)phenyl)-7-methoxy-6-(3-morpholinopropoxy)quinazolin-4-amine (compound **11q**): HR MS (ESI) *m*/*z*: calcd for C_33_H_37_N_7_O_3_ [M+H^+^]: 580.3780, found 580.3782. Yellow solid, yield 64%, Mp: 172–173 °C. ^1^H NMR (400 MHz, DMSO-d6) δ 9.60 (s, 1H), 8.63 (s, 1H), 8.26 (s, 1H), 8.02–7.88 (m, 2H), 7.56 (d, J = 8.0 Hz, 1H), 7.45 (t, J = 8.0 Hz, 1H), 6.98 (s, 3H), 5.66 (s, 2H), 4.21 (t, J = 8.0 Hz, 2H), 3.96 (s, 3H), 3.63–3.55 (m, 5H), 2.56–2.51 (m, 5H), 2.26 (s, 6H), 2.03 –1.99 (m, 2H). ^13^C NMR (100 MHz, DMSO-d6) δ 148.80, 147.06, 140.53, 138.40, 136.27, 131.40, 130.04, 129.47, 126.17, 122.38, 122.05, 120.81, 119.33, 67.61, 66.60, 56.35, 55.43, 53.87, 53.53, 26.29, 21.30.

4-fluoro-2-((4-(3-((7-methoxy-6-(3-morpholinopropoxy)quinazolin-4-yl)amino)phenyl)-1H-1,2,3-triazol-1-yl)methyl)benzonitrile (compound **11r**): HR MS (ESI) *m*/*z*: calcd for C_32_H_31_FN_8_O_3_ [M+H^+^]: 595.3353, found 595.3357. Yellow solid, yield 58%, Mp: 164–166 °C. ^1^H NMR (400 MHz, DMSO-d6) δ 9.61 (s, 1H), 8.70 (s, 1H), 8.27 (s, 1H), 8.08–7.90 (m, 3H), 7.58–7.40 (m, 5H), 5.89 (s, 2H), 4.23–4.21 (m, 3H), 3.97 (s, 3H), 3.60–3.58 (m, 5H), 2.54–2.50 (m, 5H), 2.03–1.99 (m, 2H). ^13^C NMR (100 MHz, DMSO-d6) δ166.25, 163.72, 156.54, 154.75, 148.82, 147.09, 142.60, 142.51, 140.56, 137.00, 136.90, 131.16, 129.51, 122.68, 122.55, 120.88, 119.41, 117.97, 117.73, 117.45, 117.23, 116.78, 108.48, 108.44, 103.43, 67.61, 66.60, 56.36, 55.43, 53.87, 51.42, 26.30.

N-(3-(1-(3,5-dimethoxybenzyl)-1H-1,2,3-triazol-4-yl)phenyl)-7-methoxy-6-(3-morpholinopropoxy)quinazolin-4-amine (compound **11s**): HR MS (ESI) *m*/*z*: calcd for C_33_H_37_N_7_O_5_ [M+H^+^]: 612.3712, found 612.3714. Yellow solid, yield 55%, Mp: 167–170 °C. ^1^H NMR (400 MHz, DMSO-d6) δ 9.61 (s, 1H), 8.65 (s, 1H), 8.26 (s, 2H), 7.90 (s, 1H), 7.57–7.45 (m, 4H), 6.54–6.48 (m, 3H), 5.57 (s, 2H), 4.21–3.98 (m, 5H), 3.73–3.59 (m, 12H), 2.51–2.42 (m, 5H), 2.02 –1.99 (m, 2H). ^13^C NMR (100 MHz, DMSO-d6) δ 161.21, 150.02, 148.88, 147.09, 140.54, 138.53, 131.39, 129.50, 122.42, 122.12, 120.83, 119.35, 106.59, 100.11, 67.62, 66.60, 56.39, 55.74, 55.42, 53.86, 53.54, 51.19, 26.29.

N-(3-(1-(3-iodobenzyl)-1H-1,2,3-triazol-4-yl)phenyl)-7-methoxy-6-(3-morpholinopropoxy)quinazolin-4-amine (compound **11t**): HR MS (ESI) *m*/*z*: calcd for C_31_H_32_IN_7_O_3_ [M+H^+^]: 678.1690, found 678.2611. Yellow solid, yield 52%, Mp: 172–175 °C. ^1^H NMR (400 MHz, DMSO-d6) δ 9.60 (s, 1H), 8.68 (s, 1H), 8.27 (s, 1H), 7.95–7.90 (s, 2H), 7.79 (s, 1H), 7.73 (d, J = 8.0 Hz, 1H), 7.56 (d, J = 8.0 Hz, 1H), 7.46 (t, J = 8.0 Hz, 1H), 7.38 (d, J = 8.0 Hz, 1H), 7.21 (t, J = 8.0 Hz, 1H), 5.65 (s, 2H), 4.23–4.20 (m, 2H), 3.95 (s, 3H), 3.59–3.51 (m, 5H), 2.56–2.51 (m, 5H), 2.03 –2.00 (m, 2H). ^13^C NMR (100 MHz, DMSO-d6) δ 156.70, 154.78, 148.76, 147.17, 138.93, 137.40, 137.01, 131.47, 131.29, 129.50, 127.92, 122.41, 122.24, 120.81, 119.33, 103.32, 95.56, 67.60, 66.58, 56.34, 55.43, 53.85, 52.60, 26.27.

### 5.2. Biological Study

#### 5.2.1. Cell Culture

HepG2 and L02 cells were cultured in a humidified incubator (Thermo Scientific™ Forma™ Direct Heat CO_2_ Incubator, Waltham, MA, USA) at 37 °C with 5% CO_2_ in DMEM and RPMI-1640 supplemented with 10% fetal bovine serum (FBS) and 1% Penicillin Streptomycin.

#### 5.2.2. CCK-8 Assay and IC_50_ Measurement

HepG2 and L02 cells in the logarithmic growth phase were uniformly seeded in 96-well plates at a density of 5000 cells per well and incubated in a cell culture incubator for 24 h. For the initial screening, cells were treated with 20 compounds (**11a**–**11t**) and gefitinib at a concentration of 10 μM for 72 h. The IC_50_ was calculated after treating cells at concentrations of 0.625, 1.25, 2.5, 5, and 10 μM for 72 h. Each drug was tested in triplicate, and the experiment was independently repeated three times. After adding 10 μL of CCK-8 solution to each well, the cells were incubated in the cell culture incubator for 30 min to 1 h, and the OD value was measured at 450 nm using a microplate reader. Cell viability was calculated using the formula: (A450sample − A450blank)/(A450control − A450blank)(A450 sample − A450 blank)/(A450 control − A450 blank)(A450sample − A450blank)/(A450control − A450blank) × 100%.

#### 5.2.3. DAPI Staining to Detect Morphological Changes in Apoptosis

HepG2 cells were seeded in 48-well plates and incubated for 24 h, then treated with different concentrations of **11m** and **11t** (2, 4, and 8 μM) for 48 h. After removing the original culture medium, the cells were washed with PBS and fixed with 4% paraformaldehyde at room temperature for 20 min. After washing with PBS, the cells were stained with DAPI (10 μg/mL) in the dark at room temperature for 5 min. After washing with PBS, apoptotic morphological changes were observed using a fluorescence microscope.

#### 5.2.4. Annexin V-FITC/PI Dual Staining for Apoptosis Detection

HepG2 cells were seeded in 6-well plates at a density of 5 × 10^6^ cells/well and incubated for 24 h. They were then treated with **11m** or **11t** at concentrations of 2, 4, and 8 μM for 48 h. After digestion with trypsin without EDTA and washing with PBS, the cells were resuspended in 500 µL Binding Buffer at a concentration of 1 × 10^6^ cells/mL. Then, 5 μL of Annexin V-FITC was added, mixed well, followed by the addition of 5 μL Propidium Iodide, mixed gently, and incubated in the dark at room temperature (25 °C) for 10 min. Detection was performed using a flow cytometer (BD Accuri™ C6 Plus) within 1 h.

#### 5.2.5. Colony Formation Assay

HepG2 cells were seeded in 6-well plates at a density of 500 cells per well and incubated at 37 °C for 24 h, followed by treatment with **11m** or **11t** (2, 4, and 8 μM) for 24 h. The medium was then replaced with fresh culture medium, and after 7 days, the culture medium was removed and washed with PBS. Cells were fixed with 4% paraformaldehyde at room temperature for 20 min, stained with 0.1% crystal violet staining solution at room temperature for 20 min, and excess staining solution was washed off with distilled water. The plates were dried at 37 °C, photographed, and the number of colonies was counted using ImageJ.

#### 5.2.6. Scratch Wound Healing Assay

HepG2 cells were seeded in 24-well plates, and when the cell density reached 95%, a vertical scratch was made along the edge of the plate cover using a yellow pipette tip. After washing with PBS, images of the scratches were taken at 0 h under a microscope, and each well was treated with **11m** or **11t** (0, 2, 4, and 8 μM) and incubated in a cell culture incubator. Images were taken at 12, 24, and 48 h after the scratch was made.

#### 5.2.7. Transwell Assay

HepG2 cells were starved in serum-free Gibco DMEM for 24 h, then seeded in the upper chamber of a 24-well Transwell plate (Corning Inc., Corning, NY, USA) at a density of 5 × 10^5^ cells/mL. **11m** or **11t** was added to achieve final concentrations of 0, 2, 4, and 8 μM. The upper chamber’s total volume was 200 μL, while the lower chamber contained 600 μL of Gibco DMEM with 20% serum. After 48 h of incubation, the culture medium was washed off with PBS, and the cells were fixed with 4% paraformaldehyde at room temperature for 20 min. The cells were stained with 0.1% crystal violet staining solution at room temperature for 20 min, washed three times with PBS, and the cells that did not migrate through the membrane were wiped off with a cotton swab. Images were taken under an inverted microscope, and quantification was performed using ImageJ.

#### 5.2.8. DNA Damage Immunofluorescence Analysis

HepG2 cells were seeded in laser confocal culture dishes (Nest, Wuxi, China) and incubated for 24 h, followed by treatment with **11m** or **11t** (2, 4, and 8 μM) for 24 h. After washing with PBS, cells were fixed with 4% paraformaldehyde at room temperature for 20 min. After washing with PBS, cells were permeabilized with 0.5% Triton X-100 for 20 min, followed by further washing with PBS. Cells were then blocked with goat serum at room temperature for 1 h, followed by incubation with p-H2AX (Cell Signaling Technology, Beverly, MA, USA) at a dilution of 1:500 overnight at 4 °C. After washing with PBS, cells were incubated with Cy3 (Proteintech, Wuhan, China) at a dilution of 1:100 in the dark at room temperature for 1 h. After washing with PBS, nuclei were stained with DAPI for 5 min. Cells were observed and imaged using an oil immersion lens under a laser confocal microscope (Nikon, Tokyo, Japan).

#### 5.2.9. Western Blot Analysis

HepG2 cells were seeded in 6-well plates and incubated for 24 h. They were then treated with **11m** or **11t** at concentrations of 2, 4, or 8 μM for 48 h. The cells were harvested, and cytoplasmic and nuclear proteins were extracted using cell lysis buffer from Beyotime Biotechnology (Shanghai, China). Protein supernatants were mixed with loading buffer, denatured at 100 °C for 10 min, and quantified using BCA. Equal amounts of protein, along with molecular weight markers, were loaded onto a 12% SDS-PAGE gel and separated by electrophoresis for 2 h. The separated proteins were transferred onto NC membranes, and nonspecific binding sites on the membranes were blocked with 5% skim milk. Membranes were incubated with primary antibodies overnight at 4 °C, followed by incubation with HRP-conjugated secondary antibodies (goat anti-rabbit or goat anti-mouse IgG) at room temperature for 2 h. Protein bands were visualized on NC membranes using ECL ultra-sensitive chemiluminescence solution and a Bio-Rad gel imaging and analysis system.

#### 5.2.10. Acute Toxicity Test

Six 5-week-old, SPF-grade, healthy, male Kunming mice (31.13 ± 1.13 g) were purchased from Henan Skibest Biotechnology Co., Ltd. (Anyang, China) (License No.: SCXK (Yu) 2020-0005). They were housed at the SPF animal center of the School of Basic Medicine and Forensic Science, Henan University of Science and Technology, with normal solid food and drinking water provided, and a 12 h light–dark cycle each day. After one week of acclimatization, the mice were randomly divided into two groups: the control group and the treatment group (**11t**), which were administered a single gavage of the corresponding volume of solvent and drug (drug concentration: 800 mg/kg; drug solvent: 10% DMSO + 40% PEG300 + 5% Tween 80 + 45% saline) at a dosage of 0.1 mL/10 g. After administration, the mice’s activity was monitored daily, and their weight was recorded. After 14 days, blood was collected via the eye and the mice were euthanized by cervical dislocation. The heart, liver, spleen, lungs, and kidneys were extracted, rinsed with saline, and the surface moisture was absorbed with filter paper. The weight of all organs was measured using an electronic balance. The organ coefficient was calculated as organ weight/body weight × 100. Blood samples were left at room temperature for 1 h, then centrifuged at 2500 rpm for 20 min to measure liver function indicators GPT and GOT (Nanjing Jiancheng Bioengineering Institute, Nanjing, China). After weighing the organs, they were fixed in 4% tissue fixative for 24 h, dehydrated, cleared, embedded in paraffin, and stained with HE for microscopic observation of the tissues.

#### 5.2.11. Statistical Analyses

Data were presented as means ± SEM and performed using Graph Prim 7.0. A two-tailed Student’s *t*-test or one-way analysis of variance followed by a Student–Newman–Keuls (SNK) test were used to assess significant differences. *p* < 0.05 was considered statistically significant.

## Figures and Tables

**Table 1:** The inhibitory effects of gefitinib derivatives on HepG2 cells.

Compounds	% Cell Viability (10 μM)	Compounds	% Cell Viability (10 μM)
**11a**	7.07 ± 0.49	**11k**	14.72 ± 2.29
**11b**	59.50 ± 2.74	**11l**	5.85 ± 1.15
**11c**	37.72 ± 2.38	**11m**	5.86 ± 0.04
**11d**	36.48 ± 2.89	**11n**	7.68 ± 0.34
**11e**	7.03 ± 0.16	**11o**	8.06 ± 0.88
**11f**	5.72 ± 0.40	**11p**	17.36 ± 1.24
**11g**	6.50 ± 0.88	**11q**	7.14 ± 0.95
**11h**	6.80 ± 0.14	**11r**	9.11 ± 1.63
**11i**	70.10 ± 3.40	**11s**	22.89 ± 1.97
**11j**	47.33 ± 2.17	**11t**	5.22 ± 0.36
Gefitinib	31.03 ± 2.42		

**Table 2:** IC50 values of some of gefitinib derivatives on HepG2 cells.

Compounds	HepG2, IC_50_ (μM)	Compounds	HepG2, IC_50_ (μM)
	72 h		72 h
**11a**	5.49 ± 0.21	**11m**	3.08 ± 0.37
**11e**	4.04 ± 0.21	**11n**	6.10 ± 0.06
**11f**	5.15 ± 0.31	**11o**	5.14 ± 0.25
**11g**	5.23 ± 0.20	**11p**	10.10 ± 0.14
**11h**	3.91 ± 0.08	**11q**	5.38 ± 0.57
**11k**	6.21 ± 0.24	**11r**	6.68 ± 0.11
**11l**	4.53 ± 0.37	**11t**	3.60 ± 0.53
Gefitinib	11.90 ± 0.86		

## Data Availability

All relevant data are within the manuscript and its Supplementary Materials. The data sets used and analyzed during the current study are available from the corresponding author on reasonable request. We have presented all data in the form of Tables and Figures. 1,2,3-Triazole derivatives were synthesized in-house. Dimethylsulfoxide (DMSO) was obtained from Sigma-Aldrich (St. Louis, MO, USA). Dulbecco’s modified Eagle medium (DMEM), RPMI 1640 Medium, Fetal bovine serum (FBS) and penicillin/streptomycin were purchased from Gibco (Grand Island, NY, USA). Enhanced Cell Counting Kit-8. Annexin V-FITC/Propidium iodide (PI) staining kit and Matrigel Matrix were provided by BD Biosciences (Franklin Lake, NJ, USA).

## Supplementary Materials

The following supporting information can be downloaded at: https://www.mdpi.com/article/10.3390/molecules29225438/s1.
