# Supplementary material for: Synthesis of Novel Gefitinib-Conjugated 1,2,3-Triazole Derivatives and Their Effect of Inducing DNA Damage and Apoptosis in Tumor Cells
Source: Molecules. 2024 Nov 18;29(22):5438. doi: 10.3390/molecules29225438 (PMC11597353; doi:10.3390/molecules29225438)

Synthesis of Novel Gefitinib-Conjugated 1,2,3-Triazole Derivatives and Their Effect of Inducing DNA Damage and Apoptosis in Tumor Cells

Junfei Wu, Xu Huang, Shan Lu, Ziyi Wang, Longfei Mao * and Sanqiang Li *

College of Basic Medicine and Forensic Medicine, Henan University of Science and Technology, Luoyang 471003, China; wjf20220222@163.com (J.W.); wongxun2004@163.com (X.H.); lsyotsushi@163.com (S.L.); 18272893169@163.com (Z.W.)

* Correspondence: longfeimao1988@163.com (L.M.); sanqiangli2001@163.com (S.L.)

compound 3a: ^{1}H NMR (400 MHz, DMSO-d6) δ 9.59 (s, 1H), 8.65 (s, 1H), 8.25 (d, J = 4.0 Hz, 1H), 7.94 – 7.88 (m, 2H), 7.62 – 7.60 (m, 2H), 7.55 (d, J=8.0Hz, 1H), 7.45 (t, J=8.0Hz, 1H), 7.34 (d, J=8.0Hz, 2H), 5.66 (s, 2H), 4.21 (t, J = 4.0 Hz, 2H), 3.95 (s, 3H), 3.59 (t, J = 4.0 Hz, 4H), 2.04-1.97 (m, 3H), 1.23 (s, 3H). ^{13}C NMR (100 MHz, DMSO-d6) δ 156.73, 154.79, 148.75, 147.16, 140.54, 135.86, 132.22, 131.30, 130.70, 129.50, 122.41, 122.17, 121.94, 120.81, 119.33, 103.28, 67.60, 66.56, 56.34, 55.42, 53.83, 52.78, 31.75, 29.47, 29.04, 27.01, 26.24, 22.55.

Compound 3b: ^{1}H NMR (400 MHz, DMSO-d6) δ 9.60 (s, 1H), 8.69 (s, 1H), 8.49 (s, 1H), 8.27 (s, 1H), 7.96-7.89 (m, 3H), 7.76 (t, J=8.0Hz, 1H), 7.61 – 7.56 (m, 2H), 7.49-7.45 (m, 2H), 7.22 (s, 1H), 5.89 (s, 2H), 4.21 (t, J = 8 Hz, 2H), 3.95(s, 3H), 3.60 (t, J=8.0Hz, 4H), 2.43 (m, 4H), 2.05-2.01 (m, 2H). ^{13}C NMR (100 MHz, DMSO-d6) δ 156.79, 154.83, 148.73, 147.07, 140.56, 139.17, 134.37, 133.91, 131.20, 129.74, 122.61, 122.48, 120.85, 119.36, 117.47, 111.73, 103.25, 67.60, 66.56, 56.33, 55.42, 53.84, 51.73, 26.23.

Compound 3c: ^{1}H NMR (400 MHz, DMSO-d6) δ 9.61 (s, 1H), 8.71 (s, 1H), 8.27 (s, 1H), 8.06-7.86 (m, 3H), 7.64-7.44 (m, 5H), 5.69 (s, 2H), 4.21 (t, J = 8.0 Hz, 2H), 3.97 (s, 3H), 3.59 (t, J = 4.0 Hz, 5H), 2.51-2.49 (m, 5H), 2.03 – 1.99 (m, 2H). ^{13}C NMR (100 MHz, DMSO-d6) δ 148.83, 147.21, 140.81, 140.57, 133.73, 131.21, 130.66, 129.52, 123.22, 122.48, 122.41, 120.84, 119.37, 67.61, 66.59, 56.36, 55.42, 53.86, 52.02, 26.29.

Compound 3d: ^{1}H NMR (400 MHz, DMSO-d6) δ 9.60 (s, 1H), 8.62 (s, 1H), 8.25 (s, 1H), 8.17 – 7.68 (m, 3H), 7.55 (d, J = 4.0 Hz, 1H), 7.44 (t, J = 8 Hz, 1H), 7.29 – 7.19 (m, 5H), 5.60 (s, 2H), 4.22 – 4.19 (m, 3H), 3.98 (s, 3H), 3.59 (m, 4H), 2.50 (m, 4H), 2.31– 2.39 (m, 4H), 2.32 – 2.30 (m, 1H), 2.03 – 2.01 (m, 2H). ^{13}C NMR (100 MHz, DMSO-d6) δ 167.44, 154.87, 147.08, 140.53, 138.01, 133.47, 132.20, 132.00, 131.42, 129.81, 129.73, 129.49, 129.13, 129.97, 128.47, 128.41, 122.37, 121.96, 120.79, 119.30, 67.60, 66.57, 65.49, 56.37, 55.41, 53.83, 53.34, 30.47, 26.26, 21.17, 19.11, 13.96.

Compound 3e: ^{1}H NMR (400 MHz, DMSO-d6) δ 9.63 (s, 1H), 8.64 (s, 1H), 8.26 (s, 1H), 8.13 – 7.89 (m, 2H), 7.76 (s, 1H), 7.59 (s, 1H), 7.45 – 7.18 (m, 4H), 5.75 (s, 2H), 4.57-4.23 (m, 3H), 3.98 (s, 3H), 3.59 (m, 4H), 2.51 (m, 5H), 2.03 – 2.01 (m, 2H). ^{13}C

NMR (100 MHz, DMSO-d6) δ 160.70, 154.65, 148.96, 140.53, 137.44, 137.36, 135.24, 135.16, 131.25, 129.50, 122.56, 120.91, 119.42, 118.42, 118.18, 117.97, 67.62, 66.63, 56.39, 55.44, 53.89, 53.38, 26.32.

Compound 3f: ^{1}H NMR (400 MHz, DMSO-d6) δ 9.60 (s, 1H), 8.64 (s, 1H), 8.26 (s, 1H), 8.02 – 7.90 (m, 2H), 7.65 (d, J=8.0Hz, 1H), 7.57-7.36 (m, 4H), 5.71 (s, 2H), 4.38-4.23 (m, 3H), 3.97 (s, 3H), 3.67-3.36 (m, 5H), 2.51 (m, 5H), 2.03 – 1.99 (m, 2H). ^{13}C NMR (100 MHz, DMSO) δ 161.79, 159.29, 154.77, 148.80, 147.05, 140.55, 132.86, 132.82, 131.24, 129.48, 128.59, 128.56, 122.92, 122.76, 122.66, 122.46, 122.25, 120.83, 119.74, 119.49, 119.34, 103.38, 67.62, 66.62, 56.35, 55.44, 53.88, 47.19, 47.16, 26.31.

Compound 3g: ^{1}H NMR (400 MHz, DMSO-d6) δ 9.60 (s, 1H), 8.61 (s, 1H), 8.25 (s, 1H), 8.06 – 7.90 (m, 3H), 7.57-7.29 (m, 6H), 5.71 (s, 2H), 4.25-4.15 (m, 3H), 3.97 (s, 3H), 3.65-3.59 (m, 5H), 2.51 (m, 5H), 2.02 – 1.99 (m, 2H). ^{13}C NMR (100 MHz, DMSO) δ 161.13, 154.75, 148.81, 146.92, 140.52, 133.07, 132.98, 131.27, 130.09, 129.48, 122.50, 122.36, 120.89, 119.39, 117.68, 117.43, 115.56, 115.35, 67.62, 66.61, 56.36, 55.43, 53.87, 50.72, 26.30.

Compound 3h: ^{1}H NMR (400 MHz, DMSO-d6) δ 9.62 (s, 1H), 8.62 (s, 1H), 8.23 (s, 1H), 8.12 – 7.89 (m, 2H), 7.60-7.35 (m, 6H), 5.78 (s, 2H), 4.33-4.19 (m, 3H), 3.98 (s, 3H), 3.61-3.58 (m, 5H), 2.54-2.51 (m, 5H), 2.05 – 2.00 (m, 2H). ^{13}C NMR (100 MHz, DMSO) δ 163.11, 160.62, 148.86, 146.69, 140.48, 135.50, 135.45, 132.39, 132.29, 131.25, 129.45, 129.13, 126.39, 126.36, 122.57, 122.32, 120.96, 115.60, 115.38, 67.60, 66.56, 56.38, 55.41, 53.83, 45.09, 30.47, 26.25.

Compound 3i: ^{1}H NMR (400 MHz, DMSO-d6) δ 9.62 (s, 1H), 8.64 (s, 1H), 8.28 (s, 1H), 8.18 (d, J=8.0Hz, 1H), 8.04-7.90 (m, 1H), 7.78 (t, J=8.0Hz, 1H), 7.66 (t, J=8.0Hz, 1H), 7.58 (d, J=8.0Hz, 1H), 7.47 (t, J=4.0Hz, 1H), 7.19 (t, J=8.0Hz, 1H), 5.76 (s, 2H), 4.21-3.97 (m, 5H), 3.60-3.51 (m, 5H), 2.51-2.49 (m, 5H), 2.04 – 1.97 (m, 2H). ^{13}C NMR (100 MHz, DMSO) δ 148.83, 148.08, 147.03, 140.57, 134.93, 131.23, 131.18, 130.75, 130.20, 129.52, 125.52, 122.62, 122.87, 122.48, 120.85, 119.37, .103.44, 67.62, 66.61, 56.36, 55.43, 55.37, 53.87, 50.69, 26.30.

Compound 3j: ^{1}H NMR (400 MHz, DMSO-d6) δ 9.63 (s, 1H), 8.72 (s, 1H), 8.27 (d, J=8.0Hz, 3H), 7.91 (t, J=12.0Hz, 2H), 7.61-7.27 (m, 6H), 5.76 (s, 2H), 4.70 (s, 1H),

4.31-4.21 (m, 3H), 3.95 (s, 3H), 3.63-3.38 (m, 5H), 2.57-2.51 (m, 5H), 2.03 – 2.00 (m, 2H). ^{13}C NMR (100 MHz, DMSO-d6) δ 156.80, 154.86, 148.77, 147.75, 147.25, 143.86, 140.49, 135.94, 131.91, 131.45, 131.22, 129.52, 129.35, 124.44, 122.55, 120.50, 120.89, 119.41, 103.24, 67.61, 66.55, 56.34, 55.41, 53.83, 52.63, 49.38, 26.24.

Compound 3k: ^{1}H NMR (400 MHz, DMSO-d6) δ 9.61 (s, 1H), 8.64 (s, 1H), 8.25 (s, 2H), 7.91 (d, J=8.0Hz, 1H), 7.57 (d, J=8.0Hz, 1H), 7.47-7.40 (m, 3H), 7.29-7.25 (m, 2H), 5.73 (s, 2H), 4.36-4.20 (m, 3H), 3.99 (s, 3H), 3.61-3.59 (m, 5H), 2.54-2.51 (m, 5H), 2.04 – 2.00 (m, 2H). ^{13}C NMR (100 MHz, DMSO-d6) δ 161.83, 159.37, 154.61, 148.88, 147.00, 140.54, 131.31, 131.25, 123.33, 123.18, 122.44, 122.23, 120.84, 119.33, 116.25, 116.04, 67.60, 66.53, 56.40, 55.41, 53.81, 47.60, 47.56, 26.23.

Compound 3l: ^{1}H NMR (400 MHz, DMSO-d6) δ 9.61 (s, 1H), 8.71 (s, 1H), 8.28 (s, 1H), 8.03-7.91 (m, 2H), 7.78 (d, J=8.0Hz, 1H), 7.57 (d, J=8.0Hz, 3H), 7.47 (d, J=8.0Hz, 1H), 5.80 (s, 2H), 4.22-4.20 (m, 2H), 3.97 (s, 3H), 3.64-3.53 (m, 5H), 2.54-2.51 (m, 5H), 2.02 – 1.99 (m, 2H). ^{13}C NMR (100 MHz, DMSO-d6) δ 154.72, 148.82, 147.23, 141.13, 140.57, 131.28, 129.51, 129.12, 126.24, 126.20, 122.43, 120.82, 119.35, 67.62, 66.62, 56.35, 55.44, 53.88, 52.86, 26.31.

Compound 3m: ^{1}H NMR (400 MHz, DMSO-d6) δ 9.59 (s, 1H), 8.65 (s, 1H), 8.26 (s, 1H), 7.97-7.89 (m, 2H), 7.77 (s, 2H), 7.55-7.43 (m, 2H), 7.18 (d, J=8.0Hz, 2H), 5.63 (s, 2H), 4.31-4.22 (m, 2H), 3.96 (s, 3H), 3.64-3.57 (m, 5H), 2.73-2.57 (m, 5H), 2.05 – 2.01 (m, 2H). ^{13}C NMR (100 MHz, DMSO-d6) δ 156.74, 154.79, 148.69, 147.15, 140.54, 138.07, 136.23. 131.32, 130.72, 129.48, 122.38, 122.16, 120.80, 119.30, 103.32, 94.97, 67.54, 66.34, 56.34, 55.35, 53.67, 52.93, 31.77, 26.02.

Compound 3n: ^{1}H NMR (400 MHz, DMSO-d6) δ 9.60 (s, 1H), 8.70 (s, 1H), 8.27 (s, 1H), 7.99-7.90 (m, 2H), 7.57 (d, J=4.0Hz,1H), 7.48-7.43 (m, 5H), 7.34 (s, 1H), 5.69 (s, 2H), 4.49 (s, 1H), 4.23-4.21 (m, 2H), 3.96 (s, 3H), 3.60-3.58 (m, 5H), 2.57-2.49 (m, 5H), 2.03 –1.99 (m, 2H). ^{13}C NMR (100 MHz, DMSO-d6) δ 155.45, 154.76, 148.79, 147.19, 140.56, 138.83, 138.66, 133.81, 131.29, 131.24, 129.50, 128.67, 128.53, 128.37, 127.19, 122.42, 122.27, 120.81, 119.34, 103.36, 67.61, 66.61, 56.34, 55.44, 53.88, 53.17, 52.75, 26.31.

Compound 3o: ^{1}H NMR (400 MHz, DMSO-d6) δ 9.60 (s, 1H), 8.66 (s, 1H), 8.26 (s, 1H), 8.06-7.89 (m, 2H), 7.57-7.39 (m,7H), 5.68 (s, 2H), 4.22-4.21 (m, 2H), 3.96 (s, 3H), 3.68-3.59 (m, 5H), 2.58-2.41 (m, 5H), 2.02 –1.99 (m, 2H). ^{13}C NMR (100 MHz, DMSO-d6) δ 156.57, 154.72, 148.80, 147.16, 140.54, 135.45, 133.38, 131.32, 130.39, 129.49, 129.29, 122.41, 122.16, 120.81, 119.33, 103.40, 67.61, 66.60, 56.35, 55.43, 53.86, 52.73, 26.29.

Compound 3p: ^{1}H NMR (400 MHz, DMSO-d6) δ 9.60 (s, 1H), 8.56 (s, 1H), 8.25 (s, 1H), 8.07-7.89 (m, 2H), 7.57 (s,1H), 7.44 (d,J=8.0Hz, 1H), 7.27-7.15 (m, 4H), 5.67 (s, 2H), 4.22-4.21 (m, 2H), 4.05-3.97 (m, 3H), 3.97 (s, 3H), 3.65-3.59 (m, 5H), 2.51-2.40 (m, 5H), 2.36 (s, 3H), 2.01 –1.99 (m, 2H). ^{13}C NMR (100 MHz, DMSO-d6) δ 154.70, 148.84, 146.98, 140.53, 136.81, 134.56, 131.39, 130.93, 129.18, 128.83, 126.79, 122.41, 122.12, 120.83, 119.35, 67.62, 66.65, 60.23, 56.37, 55.45, 53.90, 51.65, 26.34, 21.23, 19.17, 14.55.

Compound 3q: ^{1}H NMR (400 MHz, DMSO-d6) δ 9.60 (s, 1H), 8.63 (s, 1H), 8.26 (s, 1H), 8.02-7.88 (m, 2H), 7.56 (d,J=8.0Hz, 1H), 7.45 (t,J=8.0Hz, 1H), 6.98 (s, 3H), 5.66 (s, 2H), 4.21 (t, J=8.0Hz, 2H), 3.96 (s, 3H), 3.63-3.55 (m, 5H), 2.56-2.51 (m, 5H), 2.26 (s, 6H), 2.03 –1.99 (m, 2H). ^{13}C NMR (100 MHz, DMSO-d6) δ 148.80, 147.06, 140.53, 138.40, 136.27, 131.40, 130.04, 129.47, 126.17, 122.38, 122.05, 120.81, 119.33, 67.61, 66.60, 56.35, 55.43, 53.87, 53.53, 26.29, 21.30.

Compound 3r: ^{1}H NMR (400 MHz, DMSO-d6) δ 9.61 (s, 1H), 8.70 (s, 1H), 8.27 (s, 1H), 8.08-7.90 (m, 3H), 7.58-7.40 (m, 5H), 5.89 (s, 2H), 4.23-4.21 (m, 3H), 3.97 (s, 3H), 3.60-3.58 (m, 5H), 2.54-2.50 (m, 5H), 2.03 –1.99 (m, 2H). ^{13}C NMR (100 MHz, DMSO-d6) δ166.25, 163.72, 156.54, 154.75, 148.82, 147.09, 142.60, 142.51, 140.56, 137.00, 136.90, 131.16, 129.51, 122.68, 122.55, 120.88, 119.41, 117.97, 117.73, 117.45, 117.23, 116.78, 108.48, 108.44, 103.43, 67.61, 66.60, 56.36, 55.43, 53.87, 51.42, 26.30.

Compound 3s: ^{1}H NMR (400 MHz, DMSO-d6) δ 9.61 (s, 1H), 8.65 (s, 1H), 8.26 (s, 2H), 7.90 (s, 1H), 7.57-7.45 (m, 4H), 6.54-6.48 (m, 3H), 5.57 (s, 2H), 4.21-3.98 (m, 5H), 3.73-3.59 (m, 12H), 2.51-2.42 (m, 5H), 2.02 –1.99 (m, 2H). ^{13}C NMR (100 MHz, DMSO-d6) δ 161.21, 150.02, 148.88, 147.09, 140.54, 138.53, 131.39, 129.50, 122.42, 122.12, 120.83, 119.35, 106.59, 100.11, 67.62, 66.60, 56.39, 55.74, 55.42, 53.86, 53.54, 51.19, 26.29.

Compound 3t: ^{1}H NMR (400 MHz, DMSO-d6) δ 9.60 (s, 1H), 8.68 (s, 1H), 8.27 (s, 1H), 7.95-7.90 (s, 2H), 7.79 (s, 1H), 7.73 (d, J=8.0Hz, 1H), 7.56 (d, J=8.0Hz, 1H), 7.46 (t, J=8.0Hz, 1H), 7.38 (d, J=8.0Hz, 1H), 7.21 (t, J=8.0Hz, 1H), 5.65 (s, 2H), 4.23-4.20 (m, 2H), 3.95 (s, 3H), 3.59-3.51 (m, 5H), 2.56-2.51 (m, 5H), 2.03 –2.00 (m, 2H). ^{13}C NMR (100 MHz, DMSO-d6) δ 156.70, 154.78, 148.76, 147.17, 138.93, 137.40, 137.01, 131.47, 131.29, 129.50, 127.92, 122.41, 122.24, 120.81, 119.33, 103.32, 95.56, 67.60, 66.58, 56.34, 55.43, 53.85, 52.60, 26.27.

3a

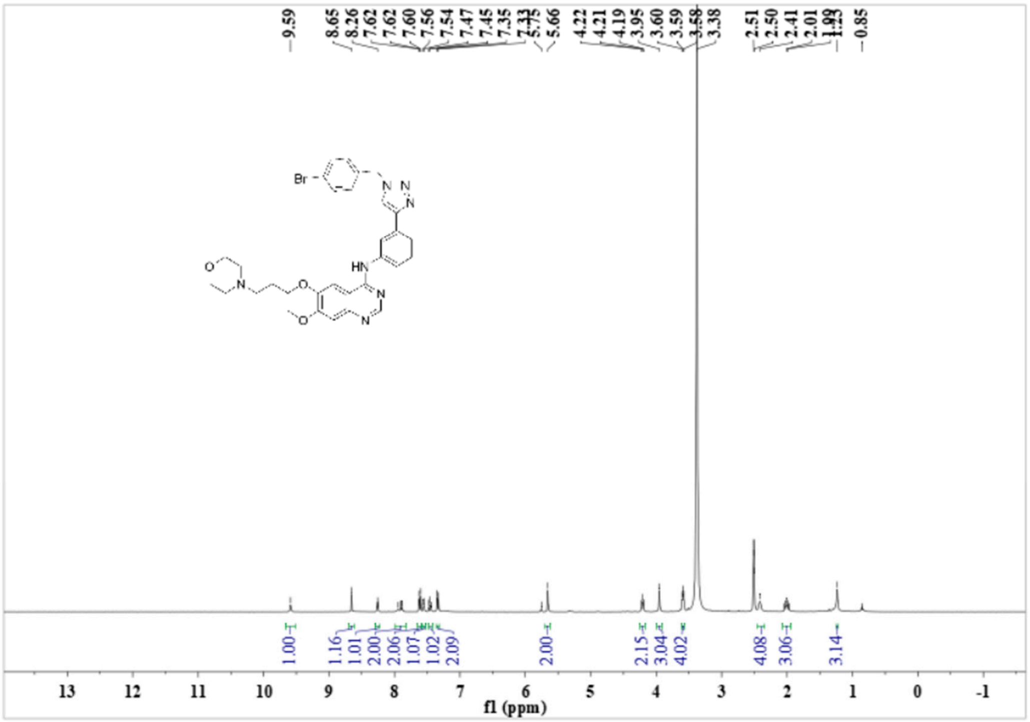
9.59
8.65
8.26
7.62
7.60
7.56
7.54
7.47
7.45
7.35
5.66
4.22
4.21
4.19
3.95
3.60
3.59
3.38
2.51
2.50
2.41
2.01
0.85
Br
N
HN
O
1.00
1.16
1.01
2.00
2.06
1.07
1.02
2.09
2.00
2.15
3.04
4.02
4.08
3.06
3.14
13 12 11 10 9 8 7 6 5 4 3 2 1 0 -1
f1 (ppm)

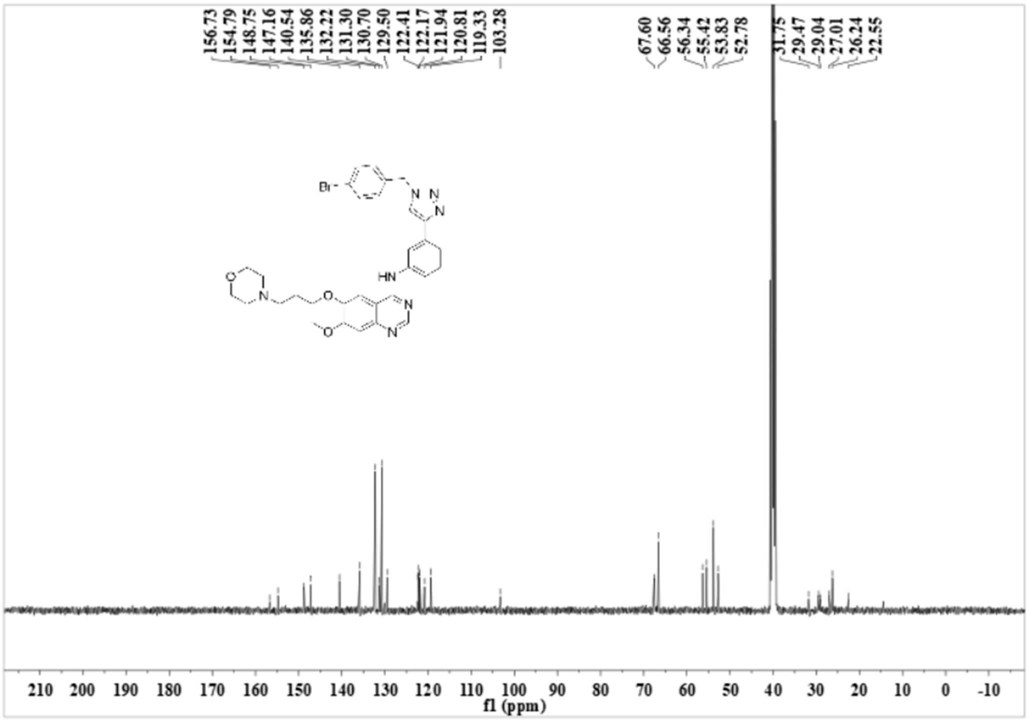
156.73
154.79
148.75
147.16
140.54
135.86
132.22
131.30
130.70
129.50
122.41
122.17
121.94
120.81
119.33
103.28
67.60
66.56
56.34
55.42
53.83
52.78
31.75
29.47
29.04
27.01
26.24
22.55
Br
N
HN
O
210 200 190 180 170 160 150 140 130 120 110 100 90 80 70 60 50 40 30 20 10 0 -10
f1 (ppm)

3b

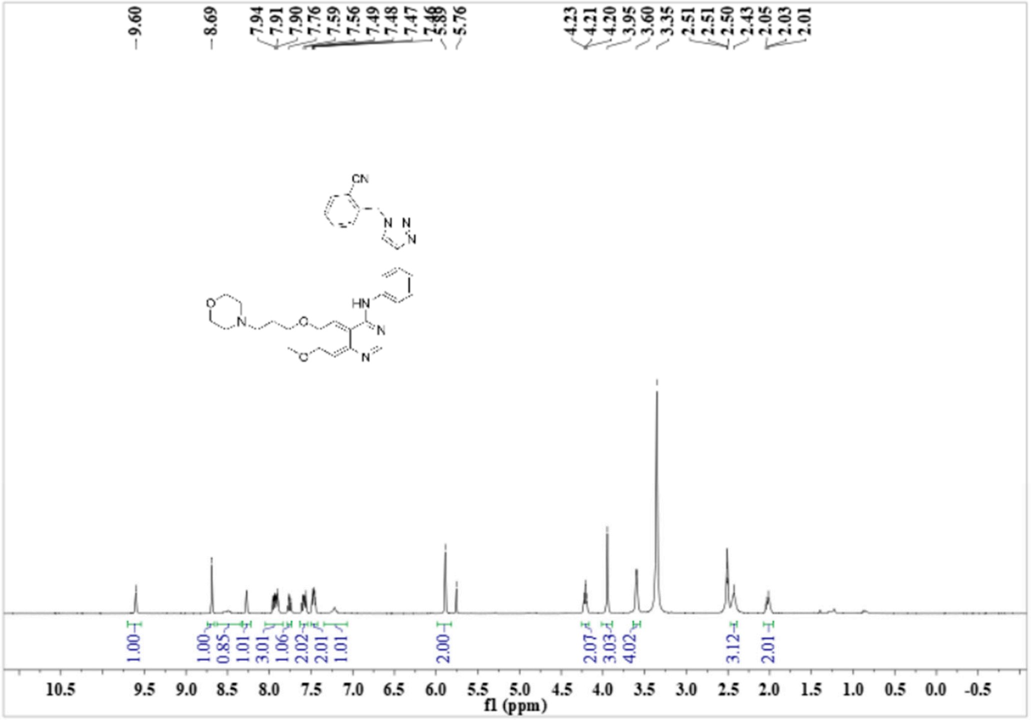
9.60
8.69
7.94
7.91
7.90
7.76
7.59
7.56
7.49
7.48
7.47
7.46
5.89
5.76
4.23
4.21
4.20
3.95
3.60
3.35
2.51
2.51
2.50
2.43
2.05
2.03
2.01
CN
HN
1.00
1.00
0.85
1.01
3.01
1.06
2.02
2.01
1.01
2.00
2.07
3.03
4.02
3.12
2.01
f1 (ppm)

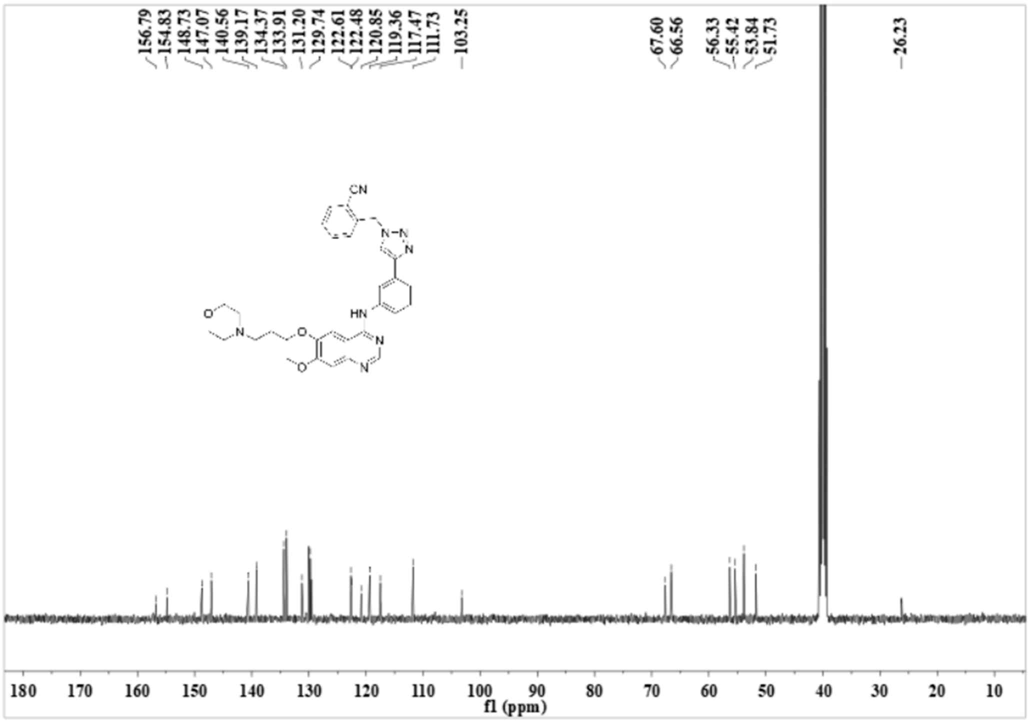
156.79
154.83
148.73
147.07
140.56
139.17
134.37
133.91
131.20
129.74
122.61
122.48
120.85
119.36
117.47
111.73
103.25
67.60
66.56
56.33
55.42
53.84
51.73
26.23
CN
HN
f1 (ppm)

3c

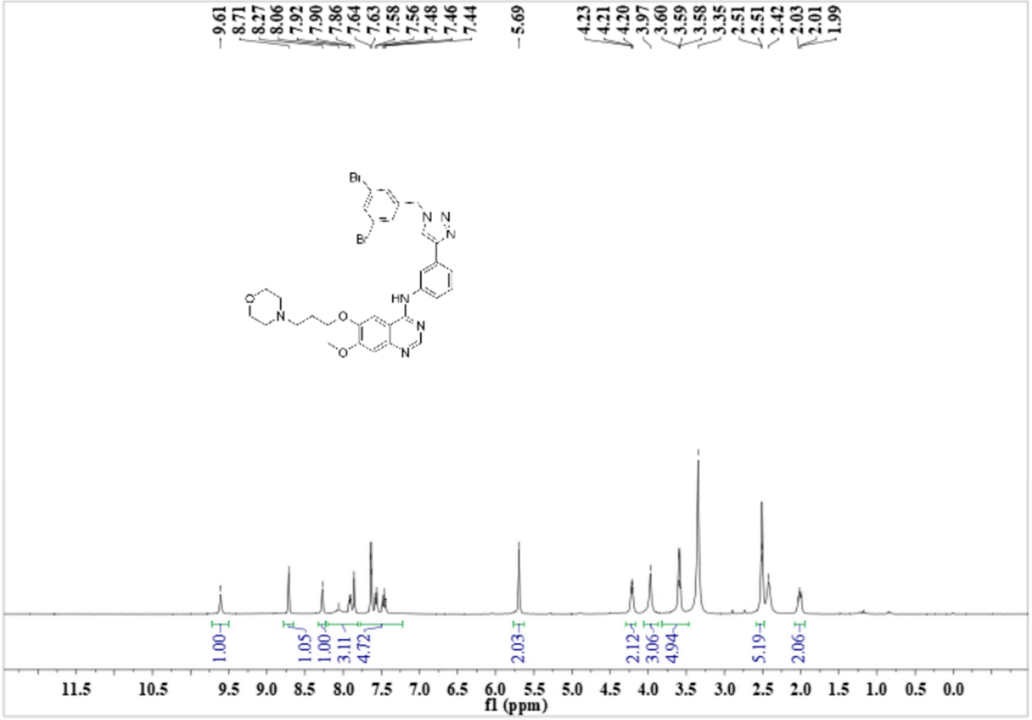
9.61
8.71
8.27
8.06
7.92
7.90
7.86
7.64
7.63
7.58
7.56
7.48
7.46
7.44
5.69
4.23
4.21
4.20
3.97
3.60
3.59
3.58
3.35
2.51
2.51
2.42
2.03
2.01
1.99
1.00
1.05
1.00
3.11
4.72
2.03
2.12
3.06
4.94
5.19
2.06
f1 (ppm)

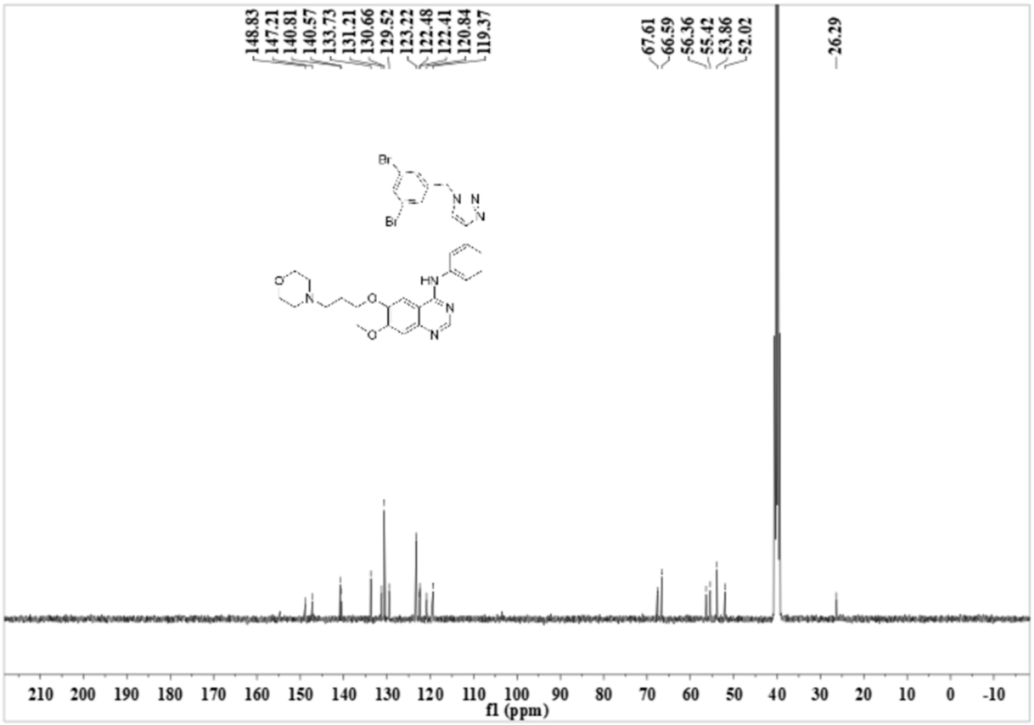
148.83
147.21
140.81
140.57
133.73
131.21
130.66
129.52
123.22
122.48
122.41
120.84
119.37
67.61
66.59
56.36
55.42
53.86
52.02
26.29
f1 (ppm)

3d

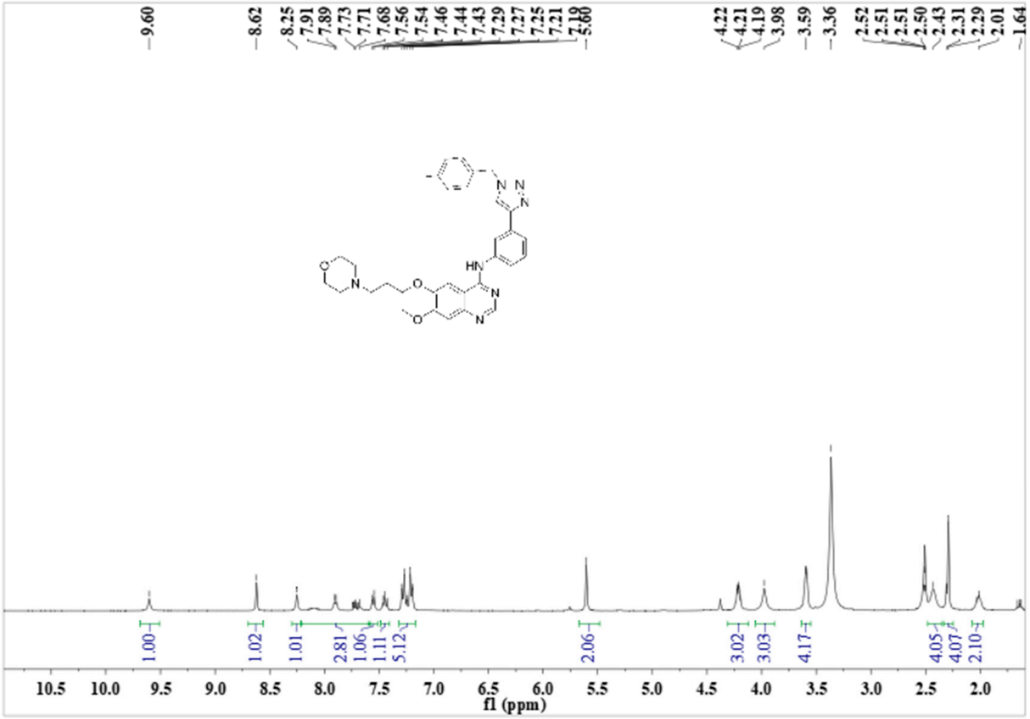
9.60
8.62
8.25
7.91
7.89
7.73
7.71
7.68
7.56
7.54
7.46
7.44
7.43
7.29
7.27
7.25
7.21
7.19
5.60
4.22
4.21
4.19
3.98
3.59
3.36
2.52
2.52
2.51
2.51
2.50
2.43
2.31
2.29
2.01
1.64
1.00
1.02
1.01
2.81
1.06
1.11
5.12
2.06
3.02
3.03
4.17
4.05
4.07
2.10
10.5 10.0 9.5 9.0 8.5 8.0 7.5 7.0 6.5 6.0 5.5 5.0 4.5 4.0 3.5 3.0 2.5 2.0
f1 (ppm)

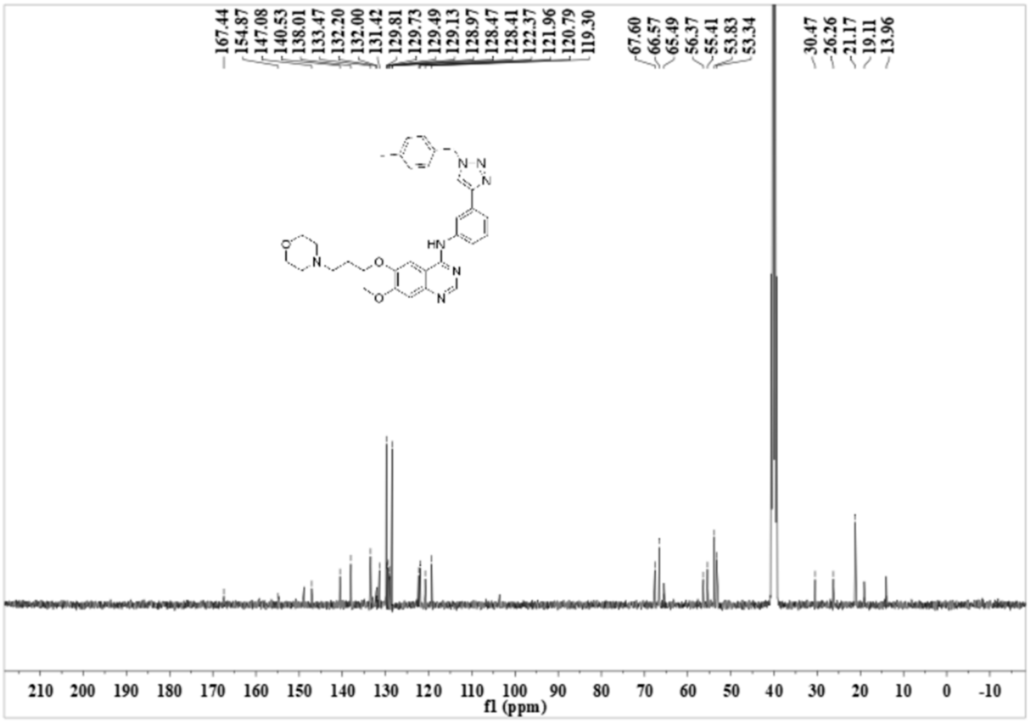
167.44
154.87
147.08
140.53
138.01
133.47
132.20
132.00
131.42
129.81
129.73
129.49
129.13
128.97
128.47
128.41
122.37
121.96
120.79
119.30
67.60
66.57
65.49
56.37
55.41
53.83
53.34
30.47
26.26
21.17
19.11
13.96
210 200 190 180 170 160 150 140 130 120 110 100 90 80 70 60 50 40 30 20 10 0 -10
f1 (ppm)

3e

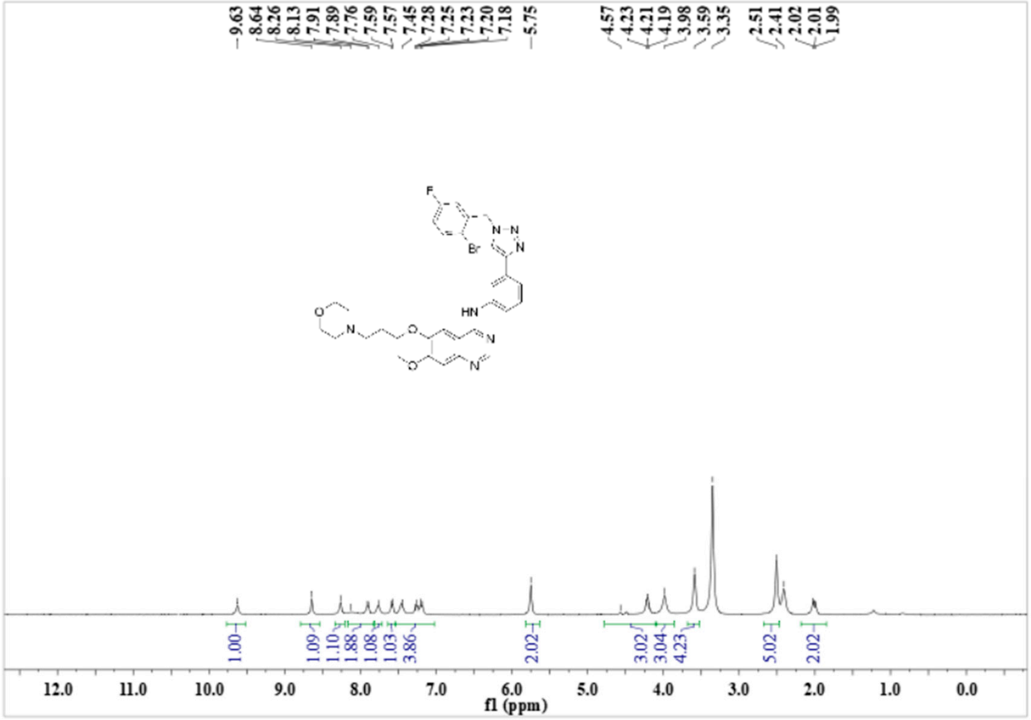
9.63
8.64
8.26
8.13
7.91
7.89
7.76
7.59
7.57
7.45
7.28
7.25
7.23
7.20
7.18
5.75
4.57
4.23
4.21
4.19
3.98
3.59
3.35
2.51
2.41
2.02
2.01
1.99
F
Br
HN
N
O
1.00
1.09
1.10
1.88
1.08
1.03
3.86
2.02
3.02
3.04
4.23
5.02
2.02
12.0 11.0 10.0 9.0 8.0 7.0 6.0 5.0 4.0 3.0 2.0 1.0 0.0
f1 (ppm)

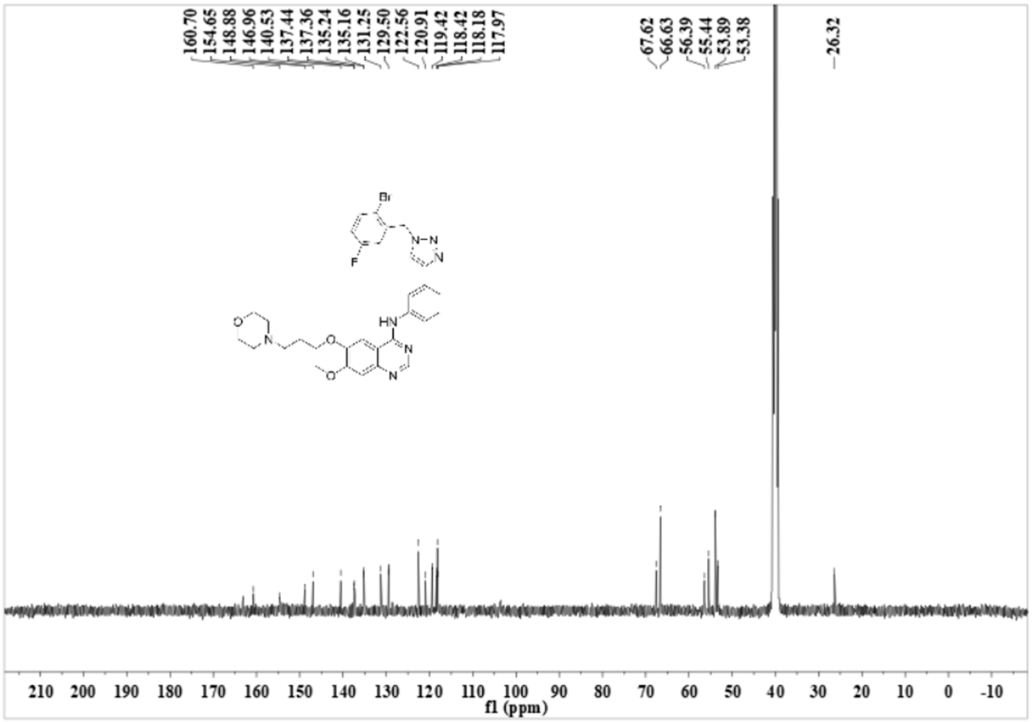
160.70
154.65
148.88
146.96
140.53
137.44
137.36
135.24
135.16
131.25
129.50
122.56
120.91
119.42
118.42
118.18
117.97
67.62
66.63
56.39
55.44
53.89
53.38
26.32
Br
F
HN
N
O
210 200 190 180 170 160 150 140 130 120 110 100 90 80 70 60 50 40 30 20 10 0 -10
f1 (ppm)

3f

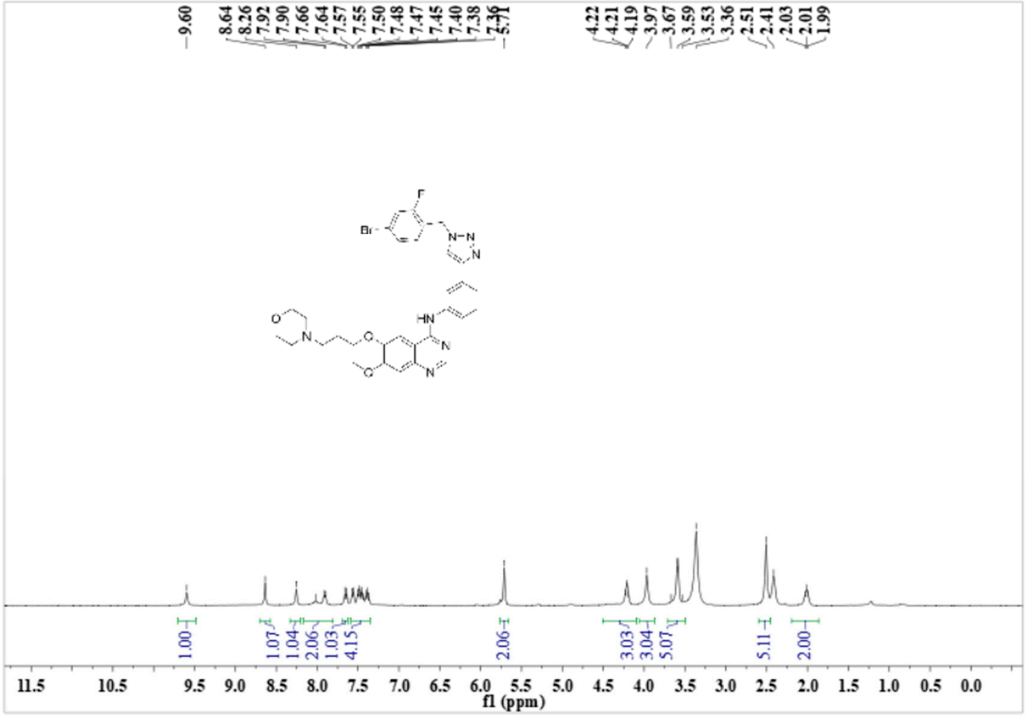
9.60
8.64
8.26
7.92
7.90
7.66
7.64
7.57
7.55
7.50
7.48
7.47
7.45
7.40
7.38
7.36
5.71
4.22
4.21
4.19
3.97
3.67
3.59
3.53
3.36
2.51
2.41
2.03
2.01
1.99
Br
F
N
N
N
HN
O
N
O
O
N
N
1.00
1.07
1.04
2.06
1.03
4.15
2.06
3.03
3.04
5.07
5.11
2.00
11.5
10.5
9.5
9.0
8.5
8.0
7.5
7.0
6.5
6.0
5.5
5.0
4.5
4.0
3.5
3.0
2.5
2.0
1.5
1.0
0.5
0.0
f1 (ppm)

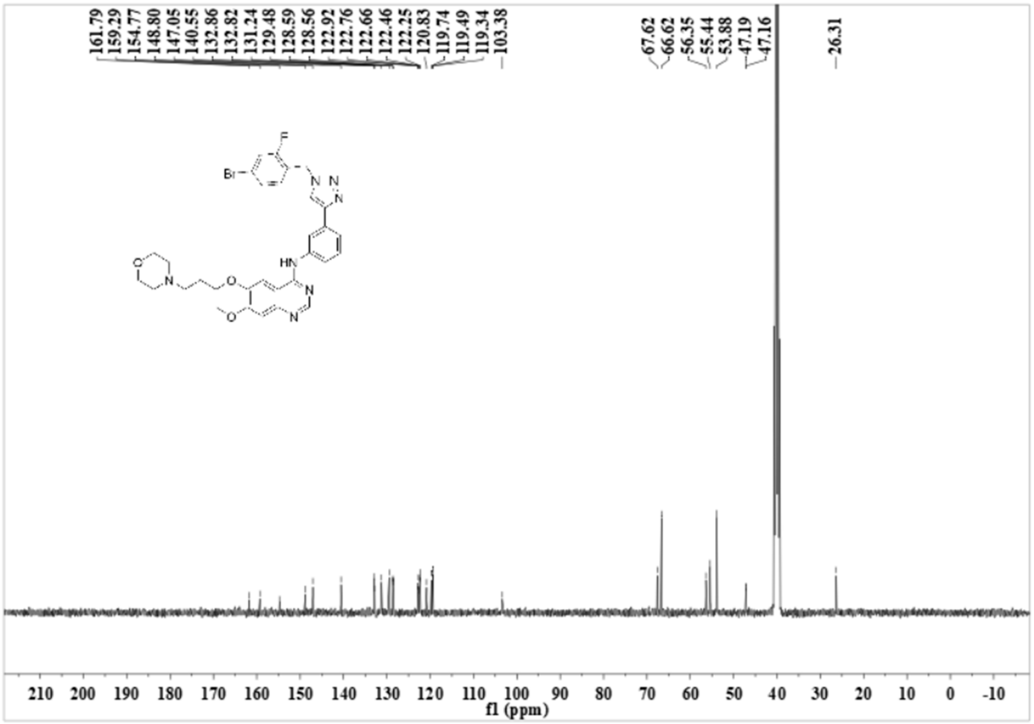
161.79
159.29
154.77
148.80
147.05
140.55
132.86
132.82
131.24
129.48
128.59
128.56
122.92
122.76
122.66
122.46
122.25
120.83
119.74
119.49
119.34
103.38
67.62
66.62
56.35
55.44
53.88
47.19
47.16
26.31
Br
F
N
N
N
HN
O
N
O
O
N
N
210
200
190
180
170
160
150
140
130
120
110
100
90
80
70
60
50
40
30
20
10
0
-10
f1 (ppm)

3g

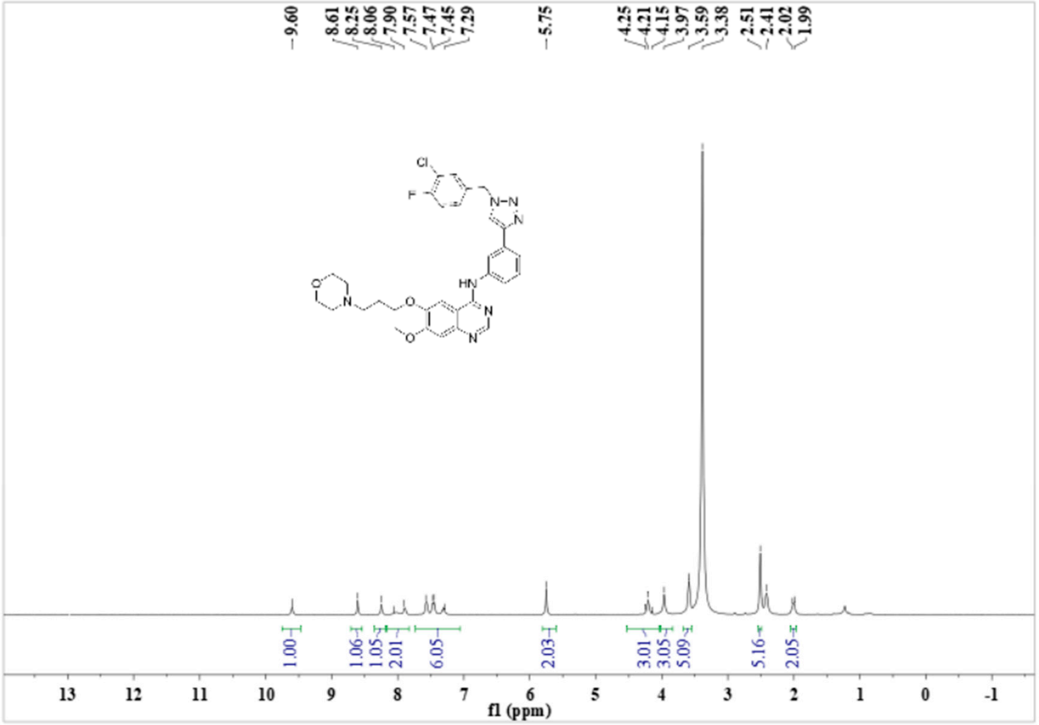
9.60
8.61
8.25
8.06
7.90
7.57
7.47
7.45
7.29
5.75
4.25
4.21
4.15
3.97
3.59
3.38
2.51
2.41
2.02
1.99
Cl
F
N
N
N
HN
O
N
O
N
N
O
1.00
1.06
1.05
2.01
6.05
2.03
3.01
3.05
5.09
5.16
2.05
13
12
11
10
9
8
7
6
5
4
3
2
1
0
-1
f1 (ppm)

3h

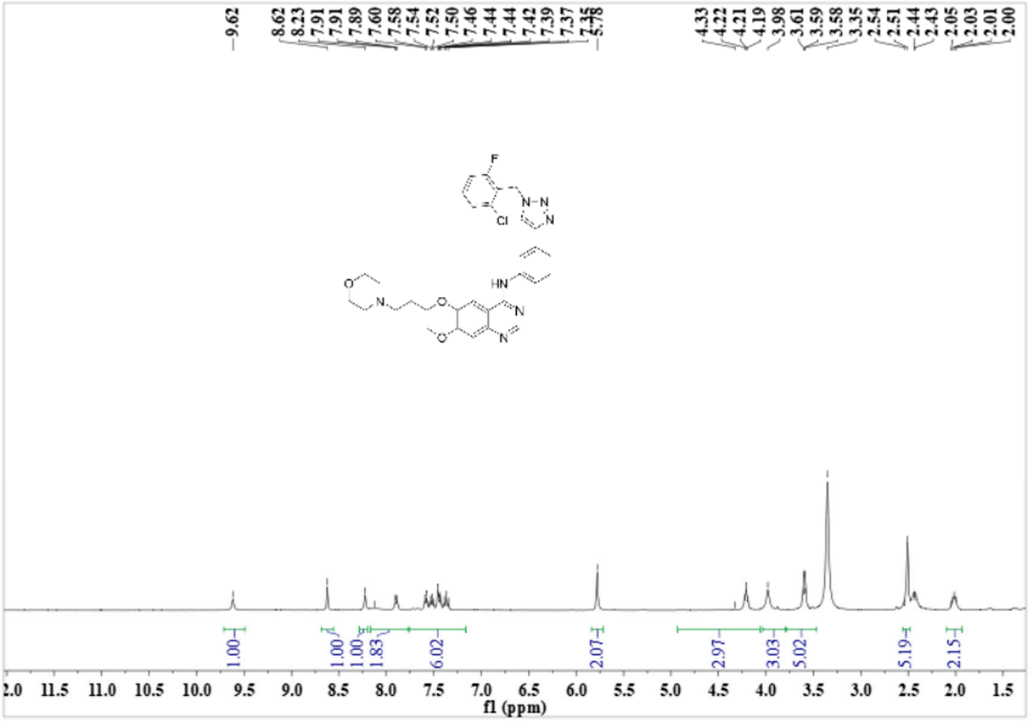
9.62
8.62
8.23
7.91
7.91
7.89
7.60
7.58
7.54
7.52
7.50
7.46
7.44
7.44
7.42
7.39
7.37
7.35
5.78
4.33
4.22
4.21
4.19
3.98
3.61
3.59
3.58
3.35
2.54
2.51
2.44
2.43
2.05
2.03
2.01
2.00
F
N
N
N
Cl
HN
O
N
O
O
N
N
1.00
1.00
1.00
1.83
6.02
2.07
2.97
3.03
5.02
5.19
2.15
fl (ppm)

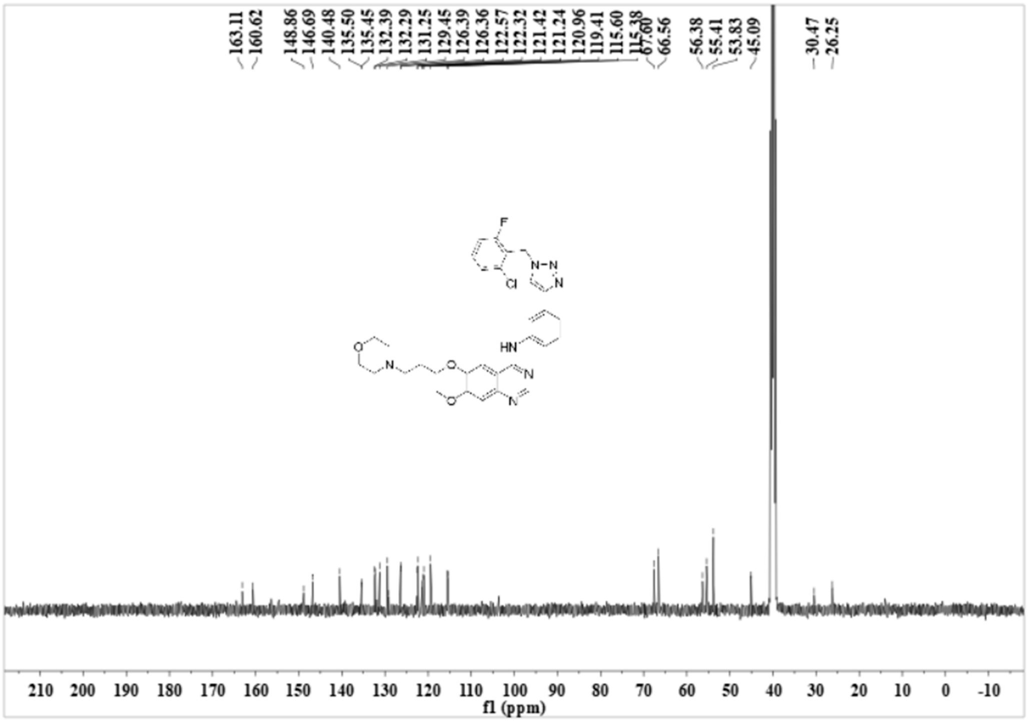
163.11
160.62
148.86
146.69
140.48
135.50
135.45
132.39
132.29
131.25
129.45
126.39
126.36
122.57
122.32
121.42
121.24
120.96
119.41
115.60
115.38
67.60
66.56
56.38
55.41
53.83
45.09
30.47
26.25
F
N
N
N
Cl
HN
O
N
O
O
N
N
fl (ppm)

3i

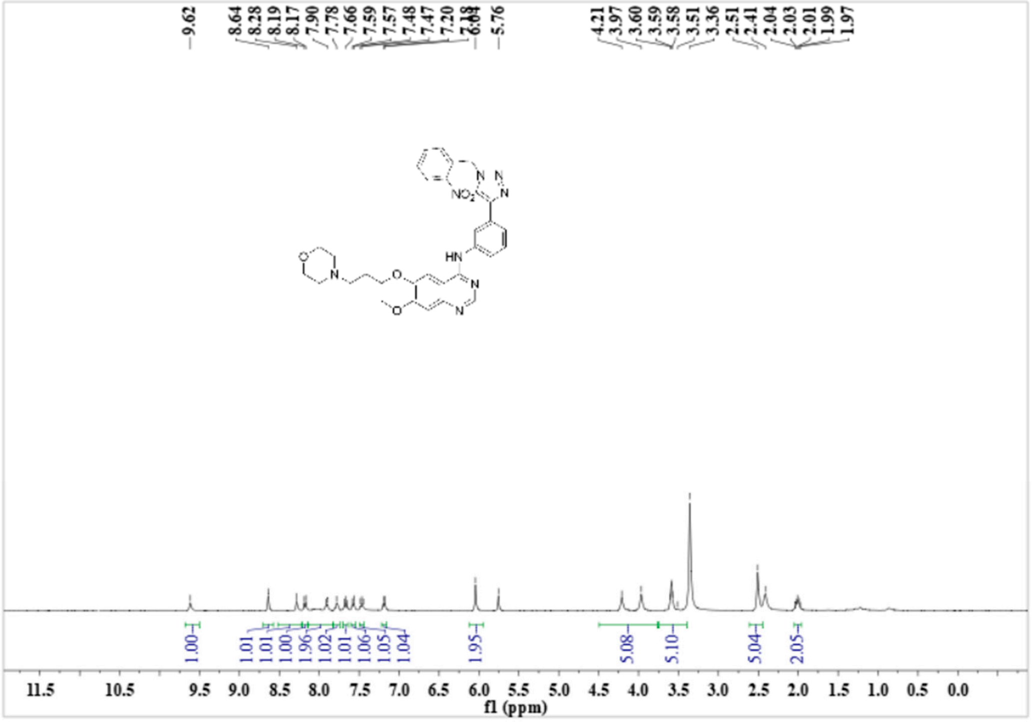

3j

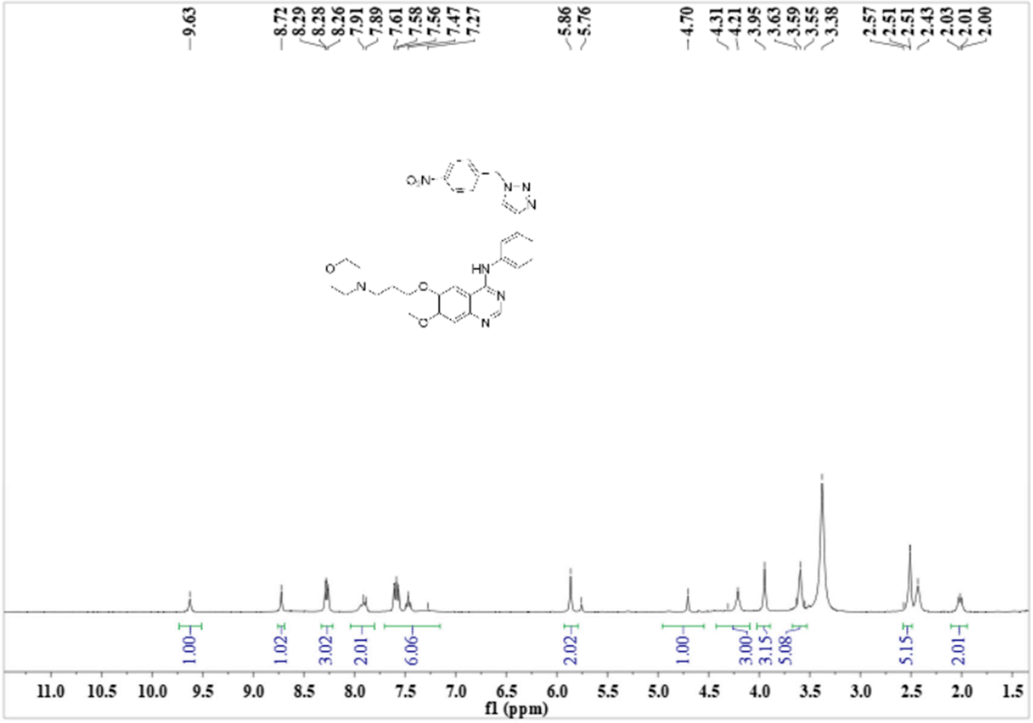

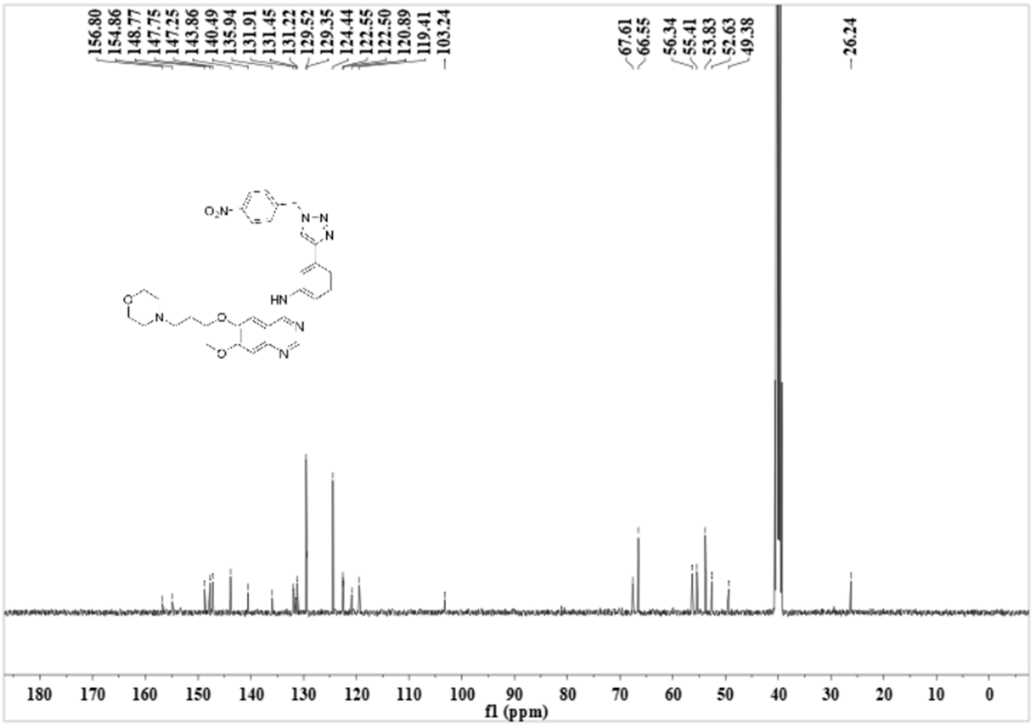

3k

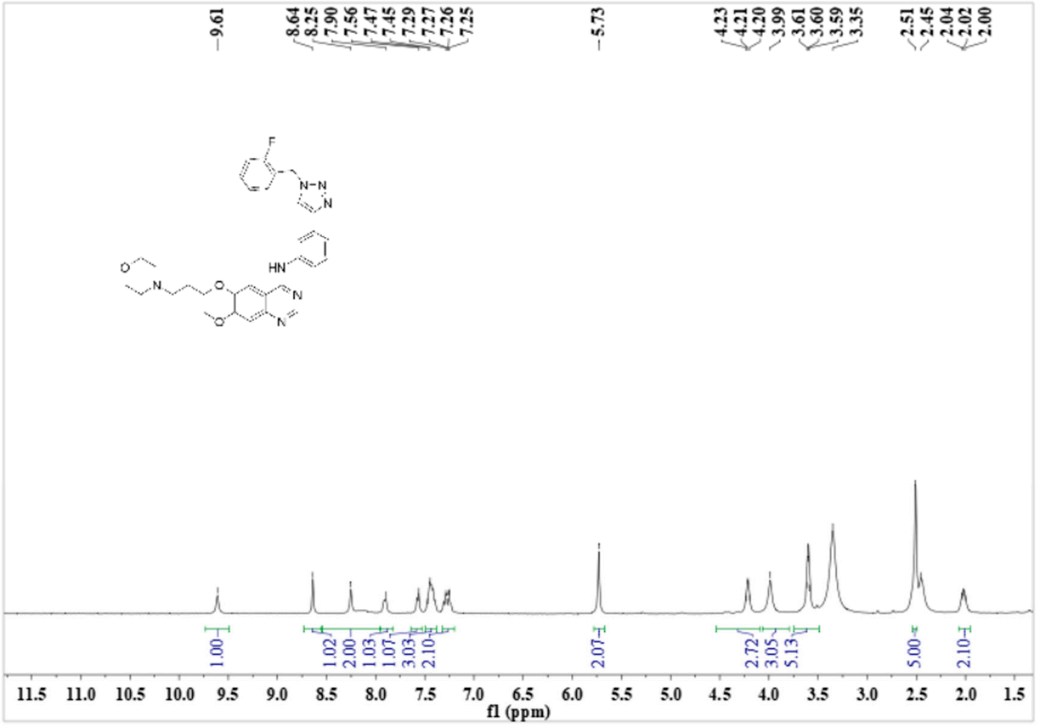

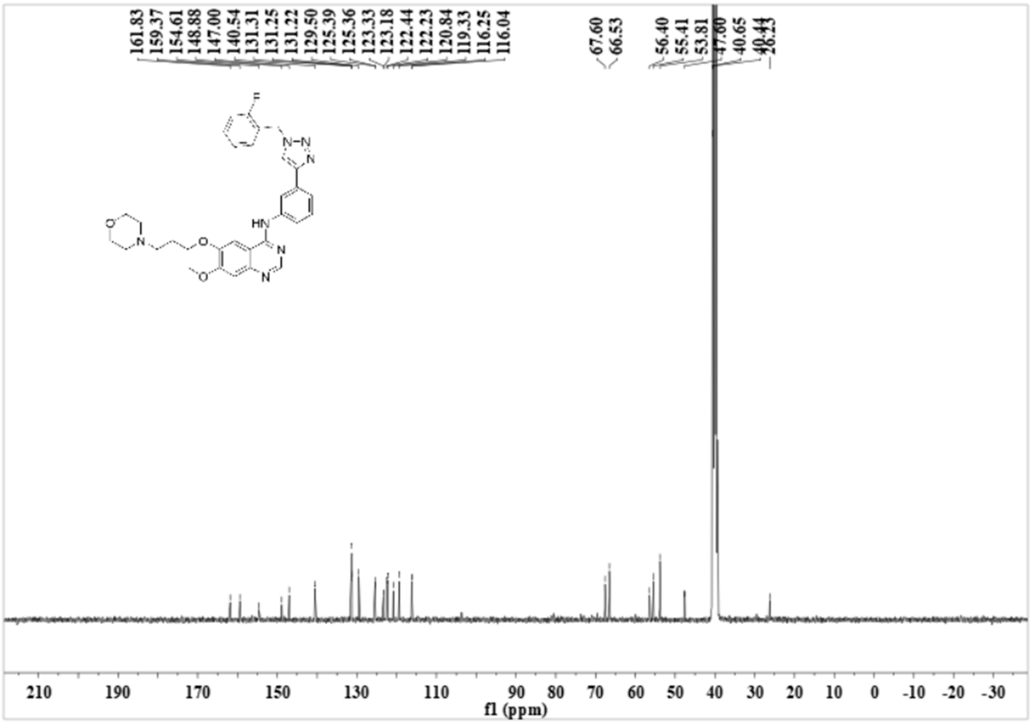

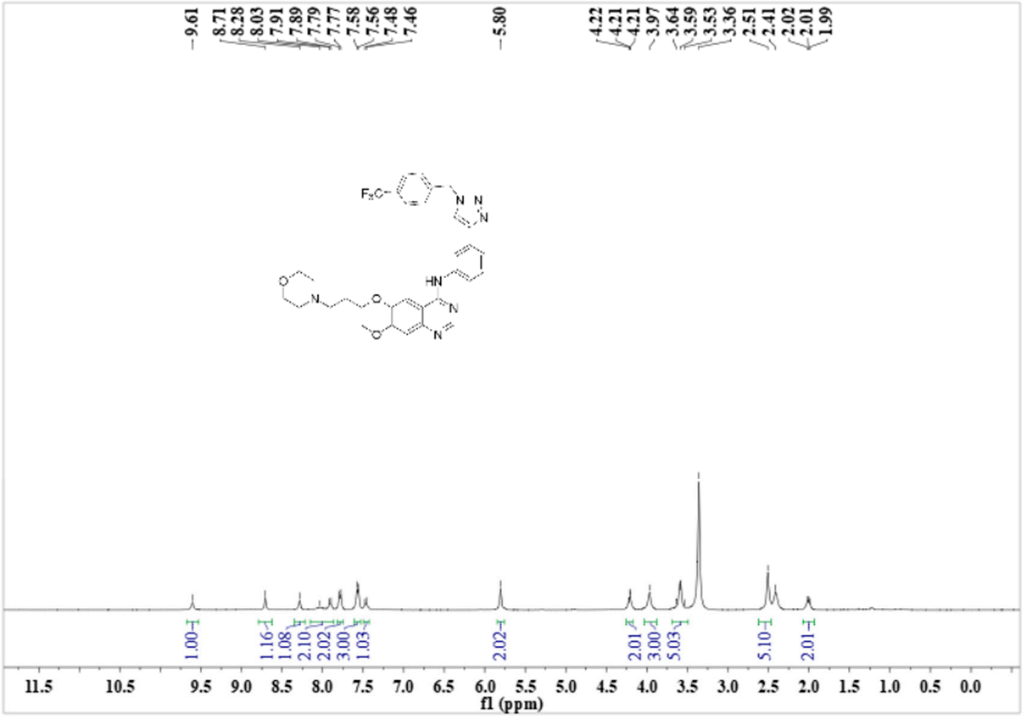

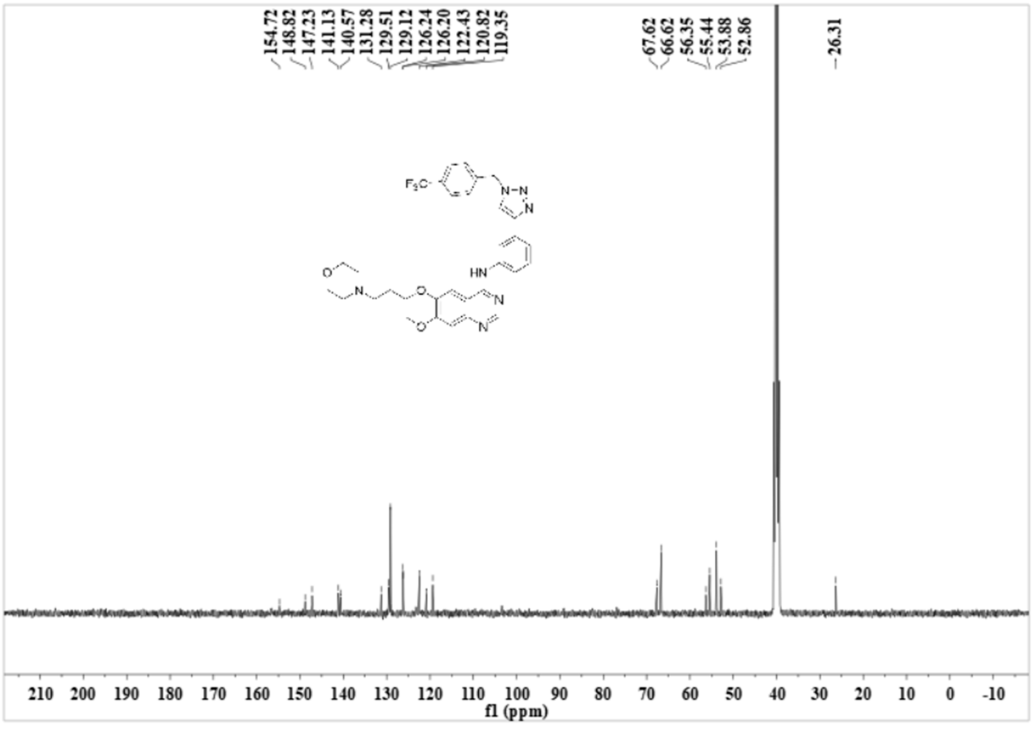

3m

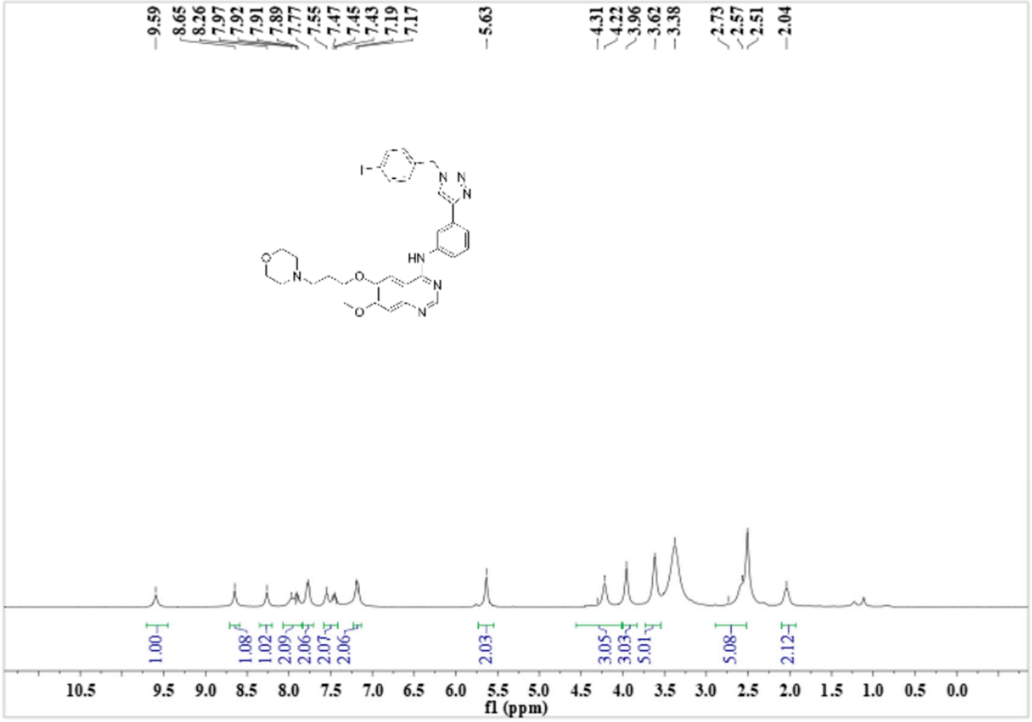

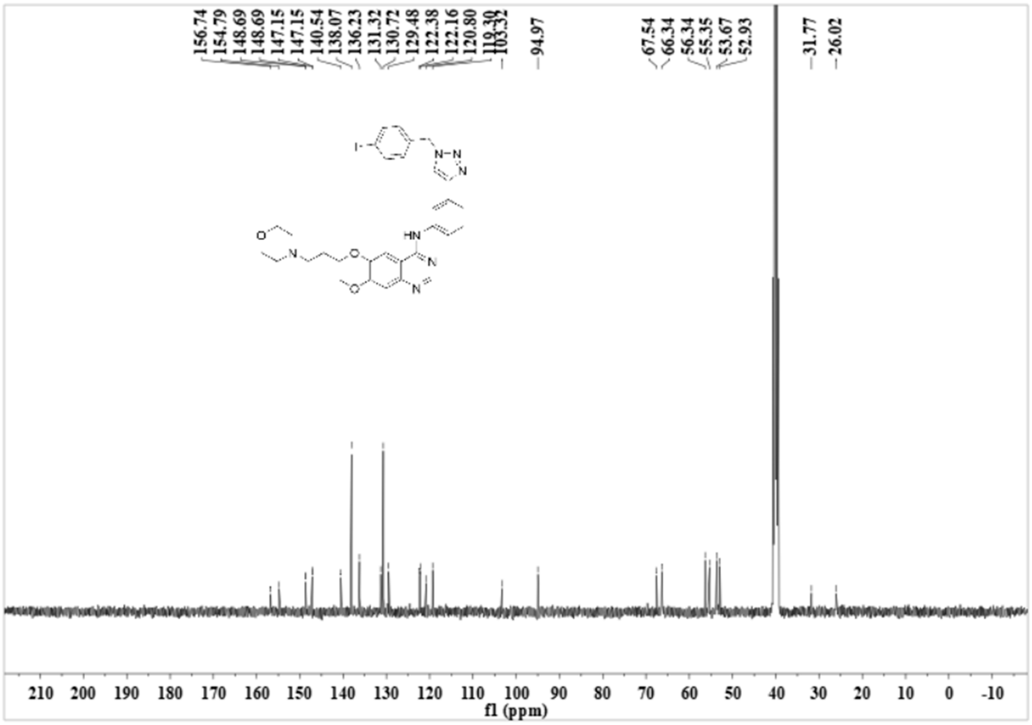

3n

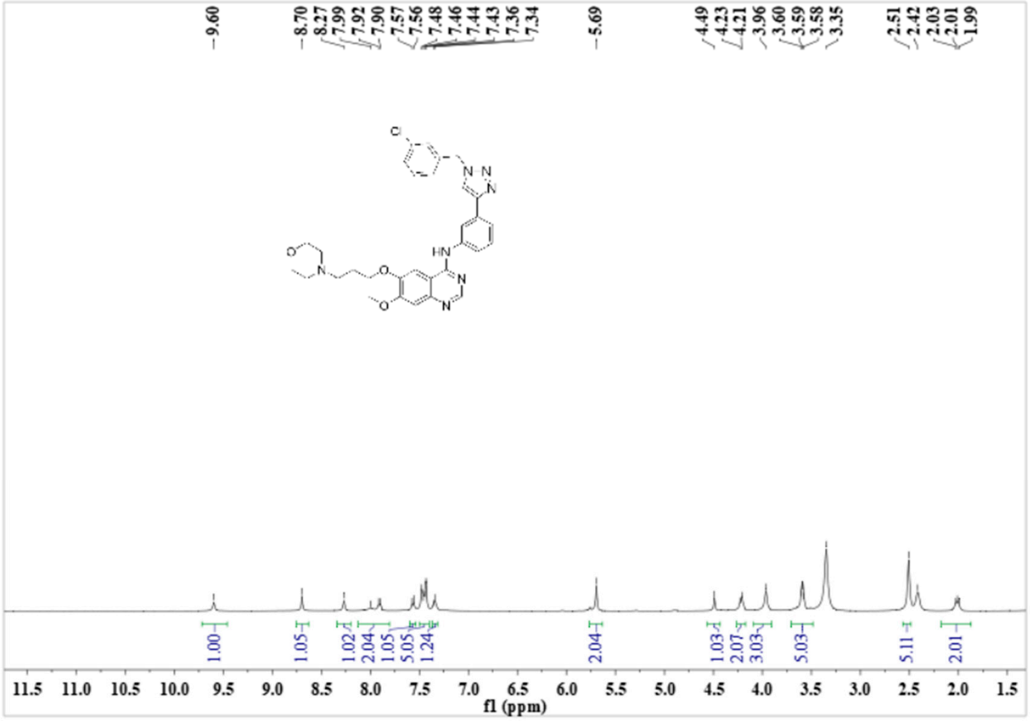

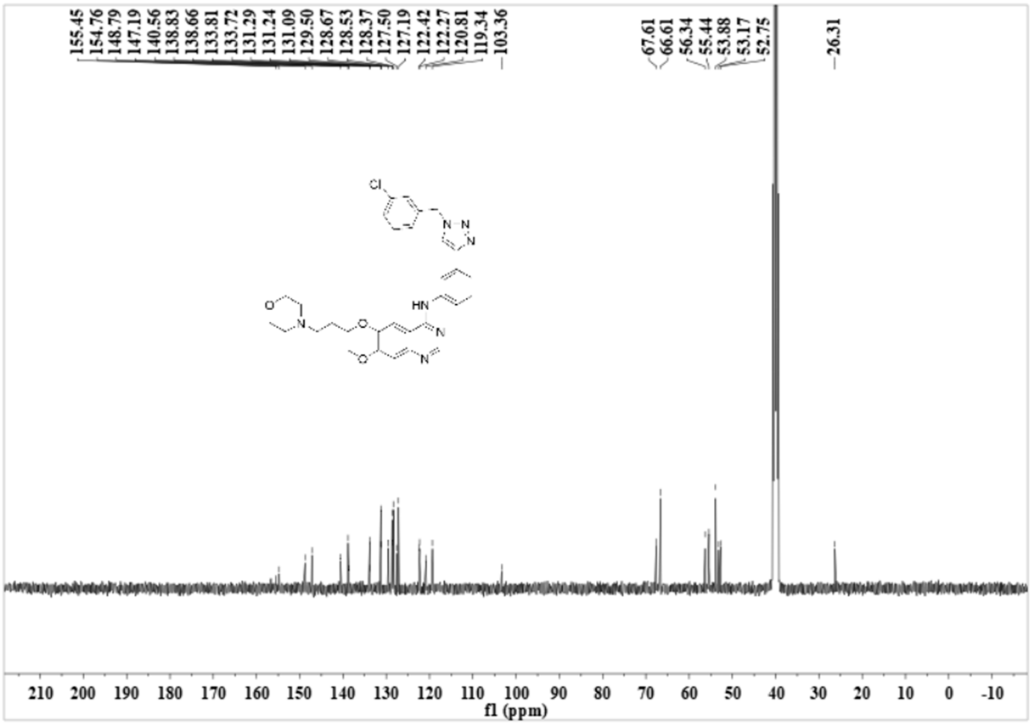

3o

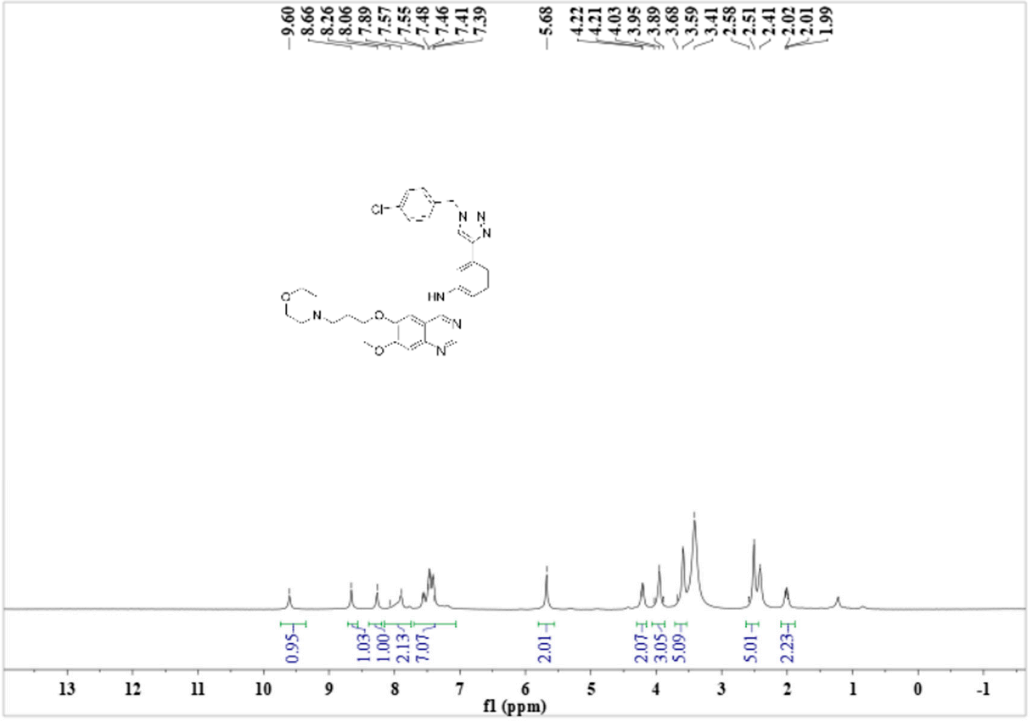

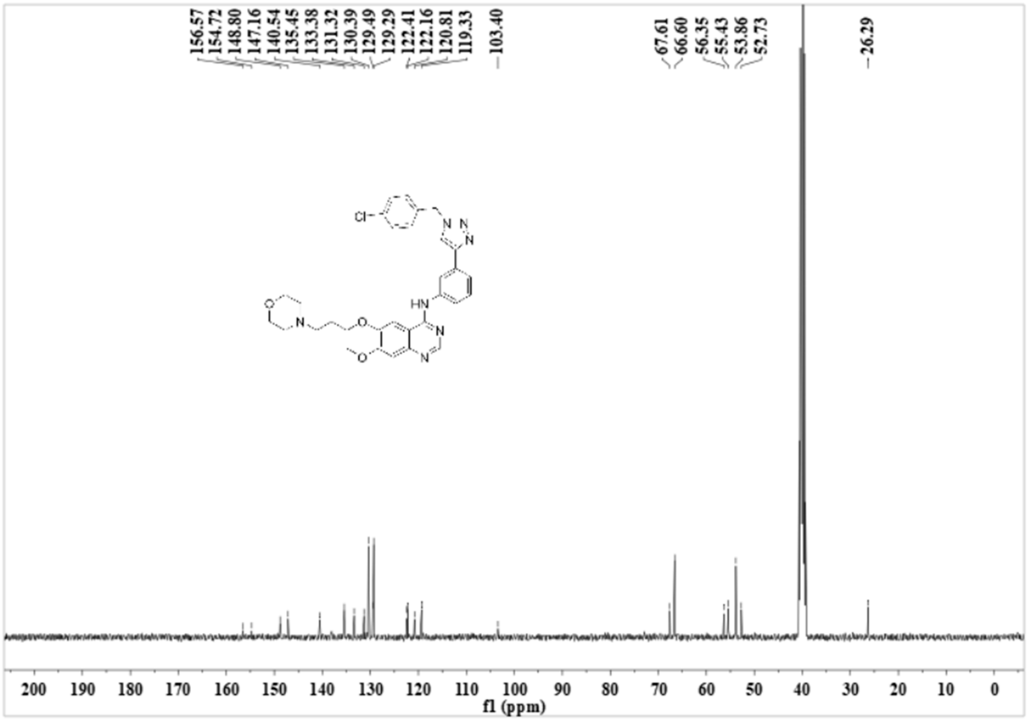

3p

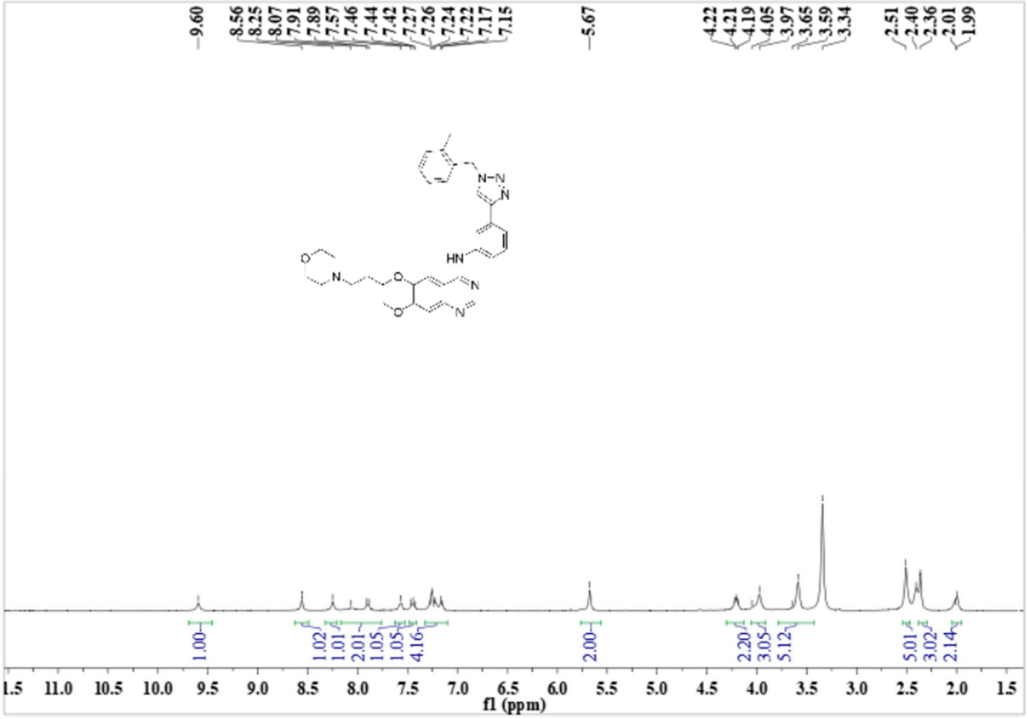

9.60
8.56
8.25
8.07
7.91
7.89
7.57
7.46
7.44
7.42
7.27
7.26
7.24
7.22
7.17
7.15
5.67
4.22
4.21
4.19
4.05
3.97
3.65
3.59
3.34
2.51
2.40
2.36
2.01
1.99
1.00
1.02
1.01
2.01
1.05
1.05
4.16
2.00
2.20
3.05
5.12
5.01
3.02
2.14
f1 (ppm)

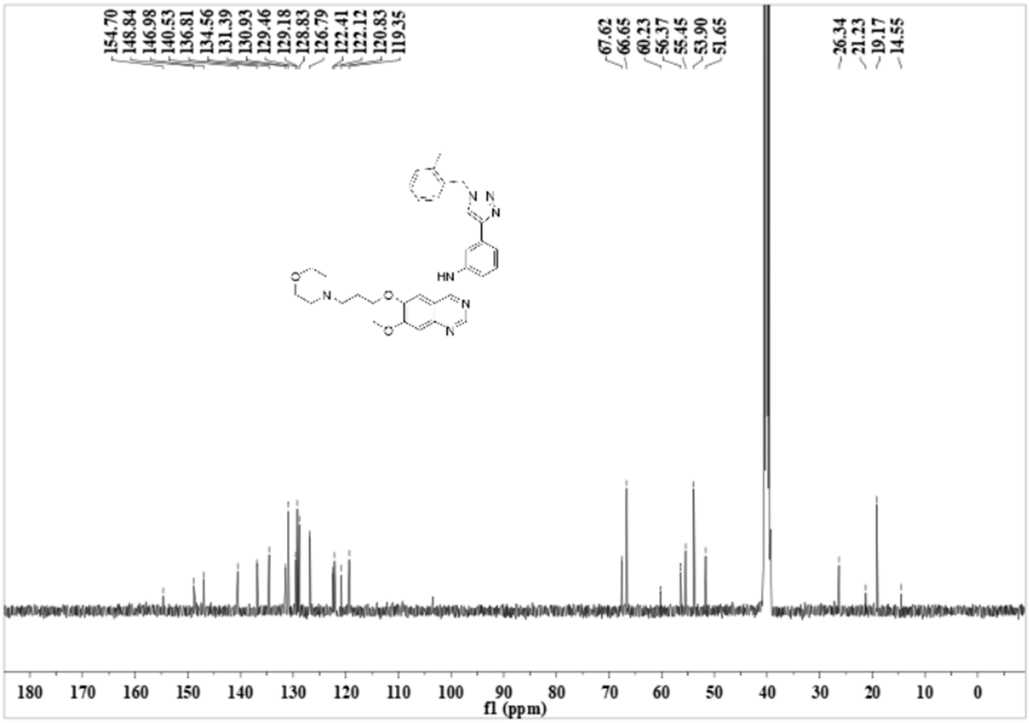

154.70
148.84
146.98
140.53
136.81
134.56
131.39
130.93
129.46
129.18
128.83
126.79
122.41
122.12
120.83
119.35
67.62
66.65
60.23
56.37
55.45
53.90
51.65
26.34
21.23
19.17
14.55
f1 (ppm)

3q

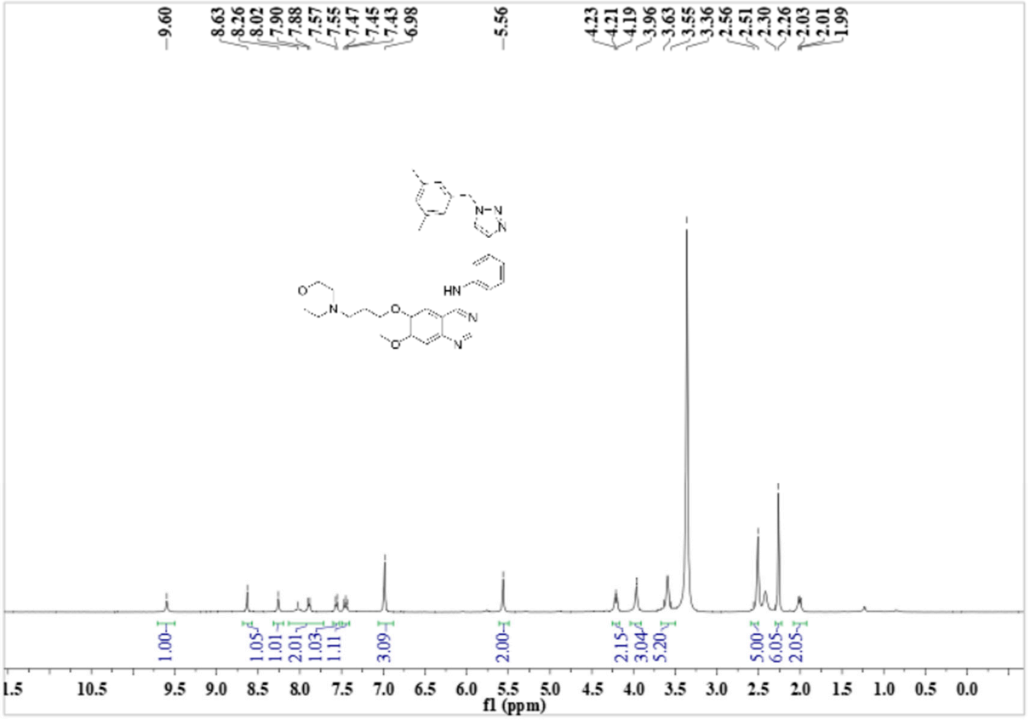

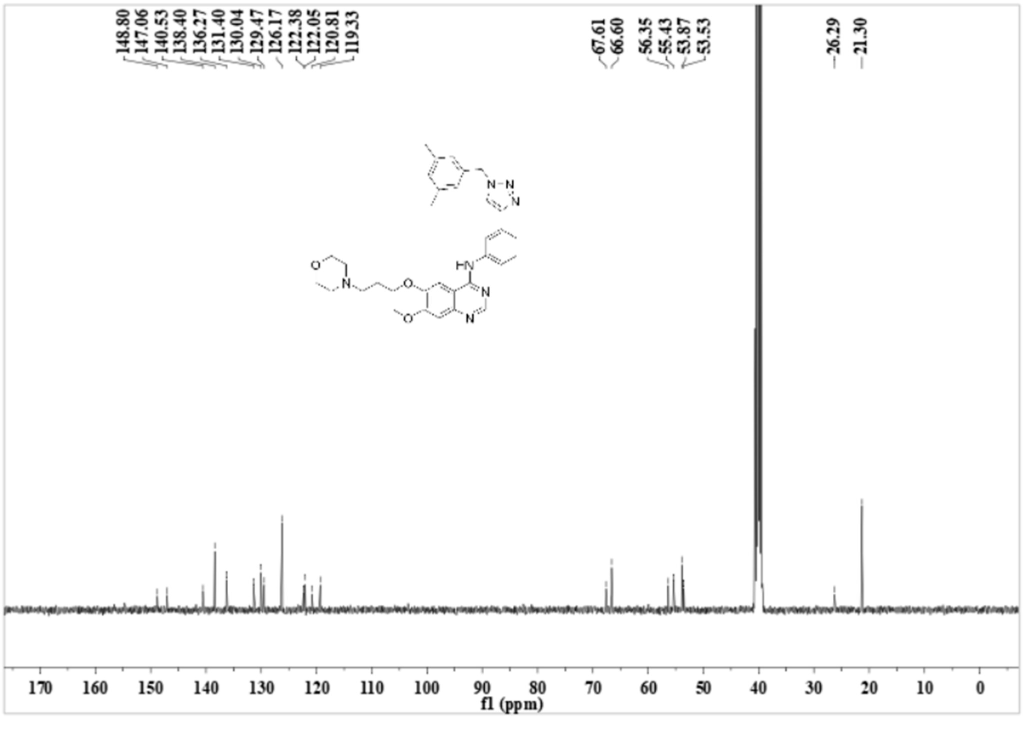

3r

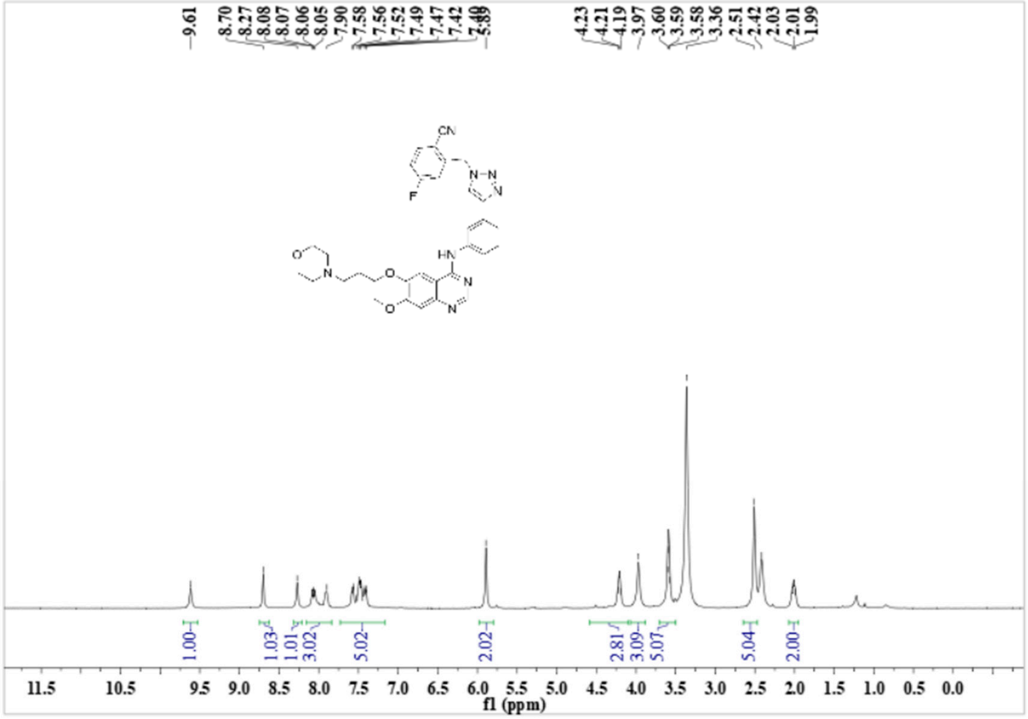

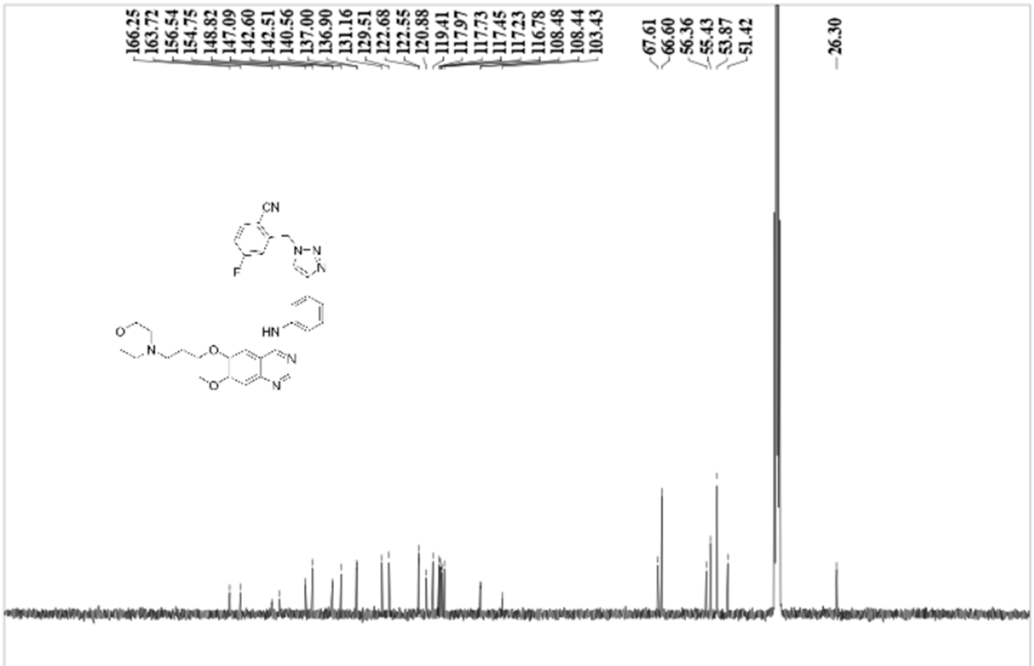

3s

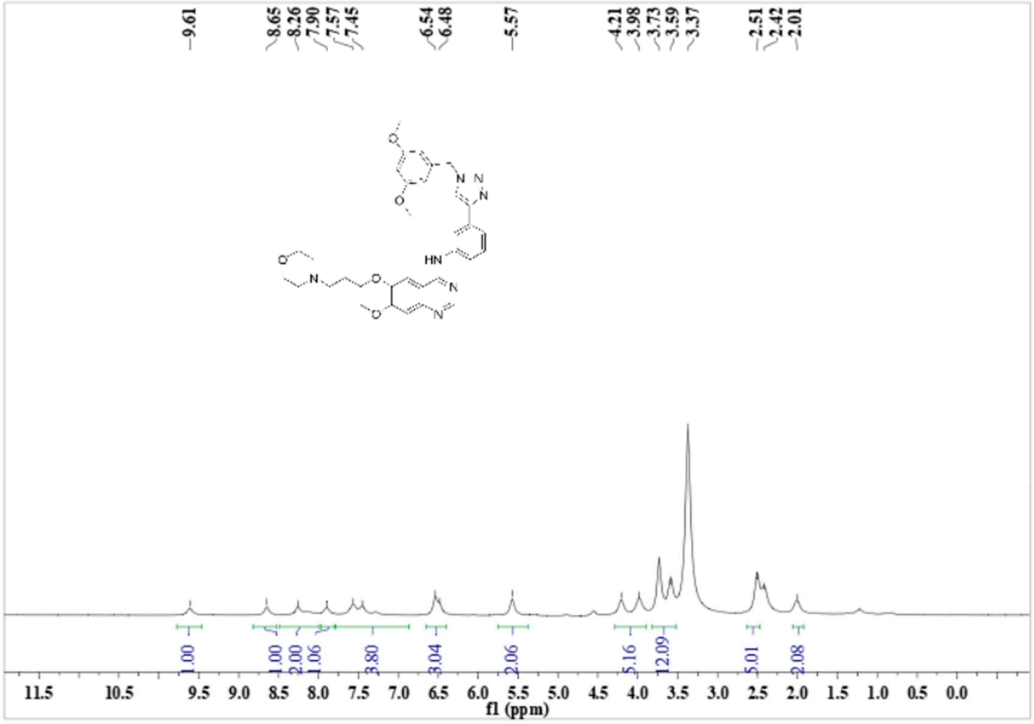
9.61
8.65
8.26
7.90
7.57
7.45
6.54
6.48
5.57
4.21
3.98
3.73
3.59
3.37
2.51
2.42
2.01
HN
N
1.00
1.00
2.00
1.06
3.80
3.04
2.06
5.16
12.09
5.01
2.08
11.5
10.5
9.5
9.0
8.5
8.0
7.5
7.0
6.5
6.0
5.5
5.0
4.5
4.0
3.5
3.0
2.5
2.0
1.5
1.0
0.5
0.0
f1 (ppm)

3t

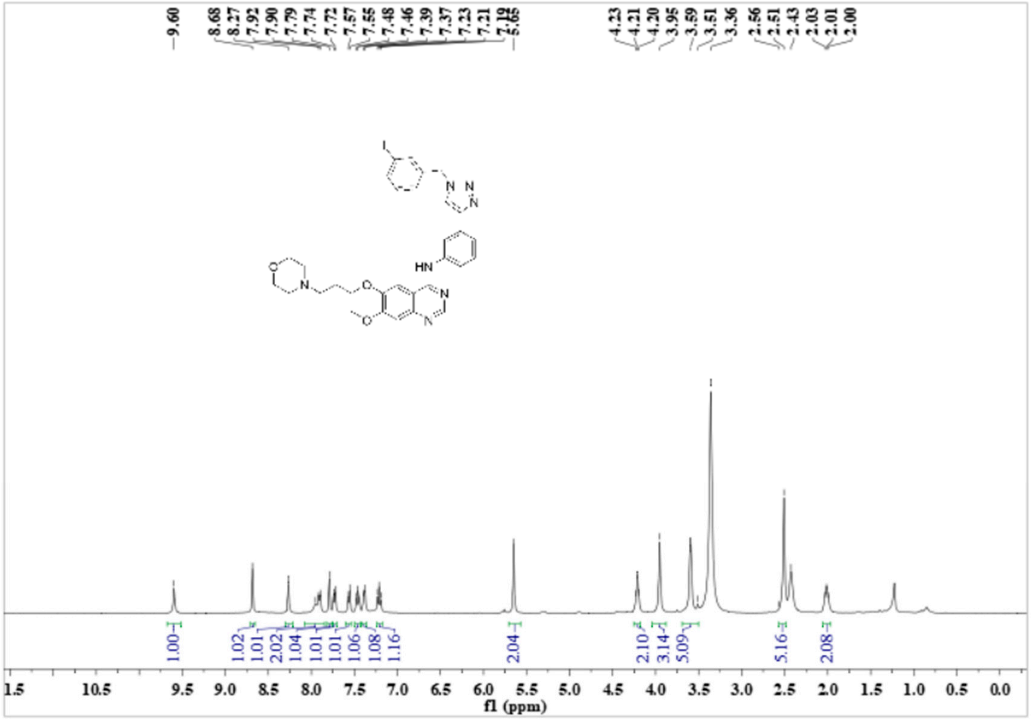

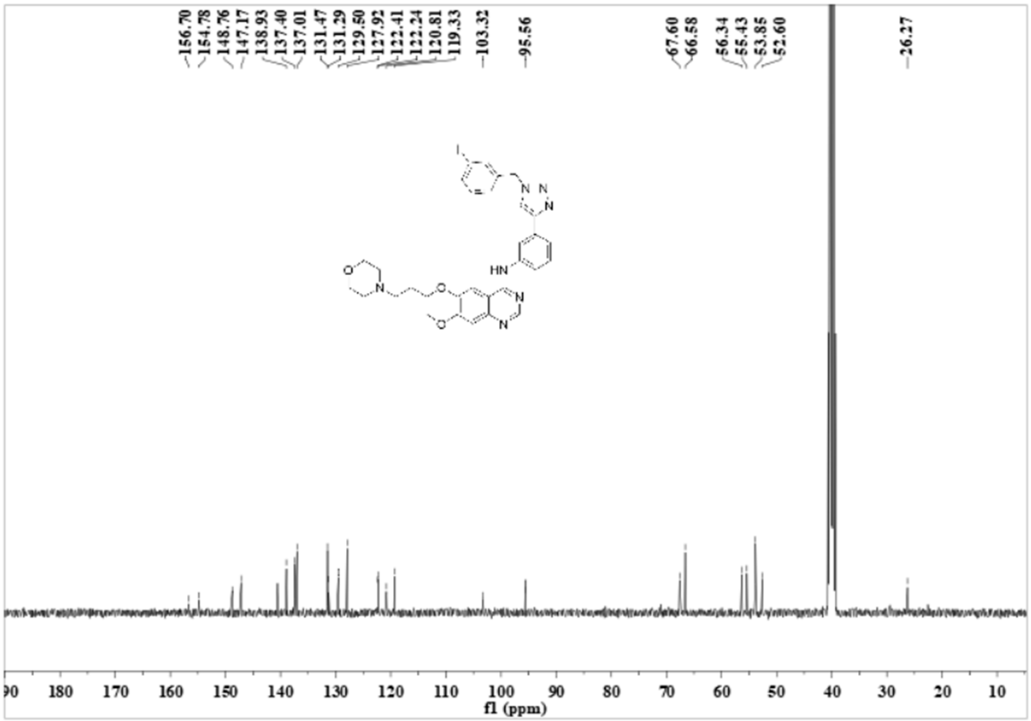

Supplement: Supplementary file 1 [file molecules-29-05438-s001.zip › molecules-3260869-supplementary.pdf]
